# Glia-to-glia serotonin signaling directs MMP-dependent infiltration for experience-dependent synapse pruning

**DOI:** 10.1371/journal.pbio.3003524

**Published:** 2025-12-01

**Authors:** Vanessa Kay Miller, Kendal Broadie

**Affiliations:** 1 Department of Biological Sciences, Vanderbilt University and Medical Center, Nashville, Tennessee United States of America; 2 Department of Cell and Developmental Biology, Vanderbilt University and Medical Center, Nashville, Tennessee, United States of America; 3 Department of Pharmacology, Vanderbilt University and Medical Center, Nashville, Tennessee, United States of America; 4 Kennedy Center for Research on Human Development, Vanderbilt University and Medical Center, Nashville, Tennessee, United States of America; 5 Vanderbilt Brain Institute, Vanderbilt University and Medical Center, Nashville, Tennessee, United States of America; University of Michigan, UNITED STATES OF AMERICA

## Abstract

Synapse connectivity is optimized in response to environmental input in critical periods, characterized by experience-dependent, temporally-restricted, and transiently-reversible synapse pruning by glial phagocytes. This precise, targeted synaptic elimination process requires glial serotonergic intercellular signaling. We discover glia-to-glia communication between different glial classes is essential for experience-dependent synaptic pruning in a well-defined *Drosophila* juvenile brain olfactory critical period. We find ensheathing glia infiltrate specific target synaptic glomeruli in response to guiding odorant experience via 5-HT_2A_ receptor (5-HT_2A_R) signaling. Using cell-targeted tryptophan hydroxylase (Trhn) RNAi to block serotonin production, we discover serotonin signaling from ensheathing glia is required for experience-dependent synapse pruning. Using cell-targeted 5-HT_2A_R RNAi, we find the serotonin receptor is required exclusively in astrocyte-like glia (ALG). Using cell-targeted 5-HT_2A_R rescue in 5-HT_2A_R null mutants, we discover the serotonin receptor mediates experience-dependent synapse pruning. Thus, glia-to-glia serotonin signaling between different glial classes mediated by 5-HT_2A_ receptors is necessary and sufficient for synapse elimination. We discover that ALG-targeted conditional 5-HT_2A_R in mature adults induces experience-dependent synapse pruning indistinguishable from the critical period mechanism. Thus, astrocyte 5-HT_2A_R signaling is sufficient to ‘re-open’ this characteristic critical period remodeling capability at maturity. We find astrocytic matrix metalloproteinase-1 (MMP-1) induced by critical period odorant experience is required for experience-dependent synapse pruning downstream of 5HT_2A_R activation. We discover that ALG-targeted MMP-1 induction restores synapse pruning in the absence of 5HT_2A_R signaling. Taken together, we conclude that glia-to-glia serotonergic 5HT_2A_R signaling drives MMP-1 for experience-dependent infiltration phagocytosis synapse pruning, and can rekindle this remodeling capacity at adult maturity.

## Abstract 2

A prior PLOS Biology study showed that experience-dependent synapse elimination in a Drosophila brain circuit depends on glial serotonergic signaling. This study shows that serotonin coordinates two distinct glial classes, with MMP-1 acting downstream of serotonin receptor activation, deepening our understanding of the mechanisms underlying brain plasticity.

## Introduction

Large-scale brain circuit remodeling driven by early-life sensory experience results from synapse connectivity refinement in transient critical periods of heightened plasticity in juveniles [1,2]. This mechanism optimizes all sensory input circuits, including visual, auditory, and olfactory modalities. Critical periods open with initial sensory experience, exhibit dynamically reversible synapse changes, and then undergo a permanent closure with consolidation of mature circuitry [2,3]. Critical period closure is presumed necessary for manifestation of mature circuit properties; however, closure prevents the correction of circuit impairments caused by trauma, injury, and disease [4,5]. This circuit connectivity remodeling occurs via opposing synapse formation and elimination mechanisms [6], with glia playing vital roles in both directions [1,7]. Overall, critical periods are characterized by net loss of synapses via experience-dependent glial phagocytosis [8–10]. In mammals, both astrocytes and microglia function as phagocytes for synapse elimination [11–13]. In *Drosophila*, comparable astrocyte-like glia (ALG), ensheathing glia (EG), and cortex glia (CG) can all act as brain phagocytes [1,14]. *Drosophila* ALG associate very closely with synapses, while EG mediate experience-dependent synapse elimination during the early-life critical period [15,16]. There is very little known about glia-to-glia signaling mechanisms orchestrating distinct glial class functions. Our goal in this study was to investigate glial subclass interactions during the mechanism of critical period experience-dependent synapse pruning.

Intercellular signaling via the biogenic monoamine serotonin (5-HT) is integral to regulation of brain circuit plasticity across multiple timescales [17,18]. Serotoninergic cells are distinguished by expression of tryptophan hydroxylase (Trhn); a rate-limiting enzyme essential for serotonin biosynthesis [19,20]. In addition to neurons, glia also employ *Trhn* to produce serotonin, and require serotonergic signaling to enable experience-dependent synapse pruning in *Drosophila* [21]. The G-protein-coupled 5-HT_2A_ receptor (5-HT_2A_R) is likewise present in both neurons and glia, playing a crucial role in regulating signaling essential for synaptic plasticity [22,23]. 5-HT_2A_Rs have long been associated with learning and memory synaptic changes underlying long-term potentiation and depression [24,25]. Importantly, the 5-HT_2A_ receptor functions in both astrocyte and microglia classes [25], and 5-HT_2A_Rs are increasingly recognized for roles in regulating the maturation and remodeling of brain circuits [26,27] *Drosophila* glia employ 5-HT_2A_R signaling to drive experience-dependent synaptic pruning during the olfactory critical period [21]. Serotonin is also implicated in many neurodevelopmental and neuropsychiatric disorders [28–30], and there is a great push to use serotonergic signaling to re-open “critical period-like” remodeling as a treatment strategy [31]. *Drosophila* glia 5-HT_2A_R expression at maturity induces de novo experience-dependent synapse elimination [21]. Our goal here was to test glial class-specific 5-HT_2A_R signaling during critical period as well as induced adult experience-driven synapse pruning.

A primary obstacle to large-scale synapse pruning following closure of the critical period is brain circuit accessibility being blocked by extracellular matrix (ECM) deposition [32,33]. Central synapses are tightly ensheathed in ECM lattices that provide foundational connectivity support for synapse stability [34,35]. Modulation of synaptic dynamics by Ca^2+^-gated ion channels is also closely regulated by ECM interactions, demonstrating vital ECM roles in synaptic plasticity and remodeling [36]. Serotonergic 5-HT_2A_R signaling regulates ECM turnover that controls synapse stability [37]. During synapse pruning, glial phagocytes must infiltrate the neuropil in a mechanism that requires remodeling of the surrounding ECM [38]. Serotonergic 5-HT_2A_R signaling activates protein kinase C (PKC), which acts downstream to increase the production of matrix metalloprotease (MMP) extracellular proteolytic enzymes that function to remodel the ECM [39]. Different MMPs degrade ECM components such as collagen and fibronectin to enable accessibility for glial infiltration during synapse pruning [40]. *Drosophila* has only two MMPs, MMP-1 and MMP-2, which drive diverse mechanisms of cellular remodeling including multiple aspects of neuronal development [32]. In a *Drosophila* injury model, glial MMP-1 activation is required for the dynamic glia responses to axotomy, including glial engulfment and the clearance of damaged axons [41]. Our goal in this study was to test MMP-1 roles in critical period synapse pruning and glial 5-HT_2A_R MMP-1 activation within the mechanism of experience-dependent synapse pruning.

Serotonergic 5-HT_2A_R signaling occurs between glia [21], but glia-to-glia class mechanisms are unknown. Here, we use a well-characterized *Drosophila* olfactory critical period [42,43] to test both serotonin production and 5-HT_2A_R function with glial class-specific transgenic drivers. We discover that EG have an essential role in serotonin synthesis for targeted synaptic neuropil infiltration and experience-dependent synapse pruning. We find an entirely distinct 5-HT_2A_R requirement in the ALG. During the juvenile critical period, we find 5-HT_2A_Rs targeted only to ALG completely rescues *5-HT*_*2A*_*R* global null mutant loss of synapse pruning, indicating ALG 5-HT_2A_R signaling fully explains the requirement in critical period experience-dependent synapse elimination. Moreover, we discover conditional 5-HT_2A_R expression in adult ALG only at maturity re-opens “critical period-like” experience-dependent synapse pruning that is indistinguishable from the juvenile brain mechanism. Downstream of 5-HT_2A_R signaling, we find that ALG MMP-1 expression is strongly induced by experience and completely required for experience-dependent synapse pruning. We discover here that MMP-1 induction targeted only to ALG completely restores synapse pruning in the absence of 5HT_2A_R signaling. Together, these results demonstrate that glia-to-glia class serotonergic 5HT_2A_R signaling activates astrocytic MMP-1 function to enable experience-dependent synapse pruning in juvenile brains, and can restore it in mature brains.

## Results

### Experience-dependent glial 5-HT_2A_ receptor signaling targets synaptic neuropil infiltration

**Fig 1:**
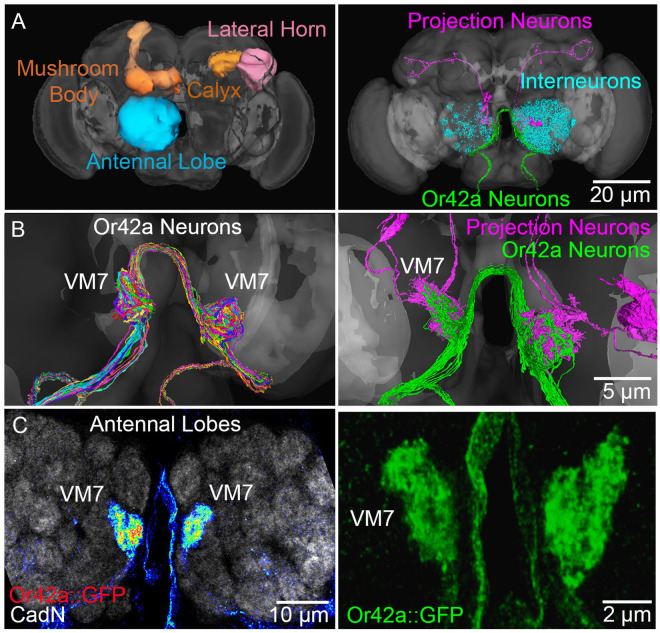
Or42a olfactory sensory neuron to projection neuron connectivity circuit. **A**, The *Drosophila* brain olfactory system synaptic neuropils; antennal lobe (AL, blue), mushroom body and calyx (MB, orange), and lateral horn (LH, pink). Right: Connectivity of the Or42a olfactory sensory neurons (green), downstream projection neurons (pink), and interneurons (blue; https://flywire.ai). **B**, The ethyl butyrate (EB) responsive Or42a neurons send axonal projections to exclusively innervate antennal lobe VM7 glomeruli. Left: The different colors show the single Or42a neuron innervation patterns. Right: Or42a neurons (green) directly synapse onto projection neurons (pink). **C**, Confocal images at lower (left) and higher (right) magnification of the Or42a neuron innervated VM7 glomeruli in the antennal lobe. Left: VM7 innervation with a direct fusion Or42a::GFP membrane marker label based on intensity (heat-map) and co-labeled with anti-N-Cadherin (CadN; gray scale) for visualization of all AL synaptic glomeruli. Right: High magnification of the Or42a::GFP membrane marker (green) to show innervation of the paired VM7 glomeruli.

The *Drosophila* olfactory circuitry (Fig 1) has a very well-defined early-life critical period [44,45], with dose-dependent and temporally-restricted activation, characterized glial phagocytes [46,47], and a highly sophisticated transgenic toolkit for the cell-specific manipulation of serotonergic signaling [48]. The juvenile brain olfactory circuit synaptic neuropils in order are the antennal lobe (AL; Fig 1A, left, blue), which houses the first synaptic relay, then the mushroom body (MB; Fig 1A, left, orange), with the postsynaptic calyx second synaptic relay, and separable projections to the lateral horn (LH; Fig 1A, left, pink), for both learned or innate olfactory behaviors, respectively [49,50]. Odorant information is received by selective olfactory sensory neuron (OSN) classes, such as the ethyl butyrate (EB) responsive Or42a receptor neurons (Fig 1A, right, green), which innervate AL glomeruli. OSNs synapse on specific projection neurons (PNs; Fig 1A, right, pink), which then relay information to the MB and LH for learning and memory. Information processing is mediated by local interneurons (Fig 1A, right, cyan), which input and receive information from OSNs and PNs to integrate signaling. EB-responsive Or42a neurons selectively innervate only the defined ventral medial 7 (VM7) glomeruli (Fig 1B, left), with densely arrayed synapses. Or42a neurons are the sole OSN class innervating the VM7 synaptic glomeruli and make precisely mapped synapses directly onto defined PNs (Fig 1B, right). This circuit connectome serves as a reference for the experiments described below.

Confocal microscopy is used to visualize Or42a neuron synaptic innervation in the AL VM7 glomeruli (Fig 1C). VM7 innervation can be visualized with a direct fusion of the Or42a promoter to green fluorescent protein (Or42a::GFP) or with Or42a-Gal4 driving the UAS-mCD8::GFP membrane marker. The Or42a OSN innervation is extremely dense, therefore, the use of heat-map visualization allows varying degrees of innervation to be separated (Fig 1C, left). These heat maps utilize fluorescence intensity of the GFP fluorophore to provide a value reflective of the degree of innervation within the neuropil. The AL is co-labeled with anti-N-Cadherin (CadN), a cell adhesion molecule, to demarcate all the synaptic glomeruli for circuit-localized studies (Fig 1C, left). Higher magnification allows for measurements of 3-dimensional innervation volumes in the VM7 synaptic glomeruli. The oval between the two brain hemisphere ALs is the esophagus opening that marks the center of the brain. The VM7 glomeruli on either side are connected with a cross-over connection (Fig 1B) on the medial aspect of the brain, which is typically not captured in our confocal imaging (Fig 1C). At higher magnification, the VM7 synaptic innervation can be visualized with a Z-stack projection (Fig 1C, right). Three-dimensional innervation volumes are measured by delineating the innervation area in each optical section, and then summing the total volume (see Methods). This approach allows for investigation of experience-dependent pruning via glial phagocytosis.

**Fig 2:**
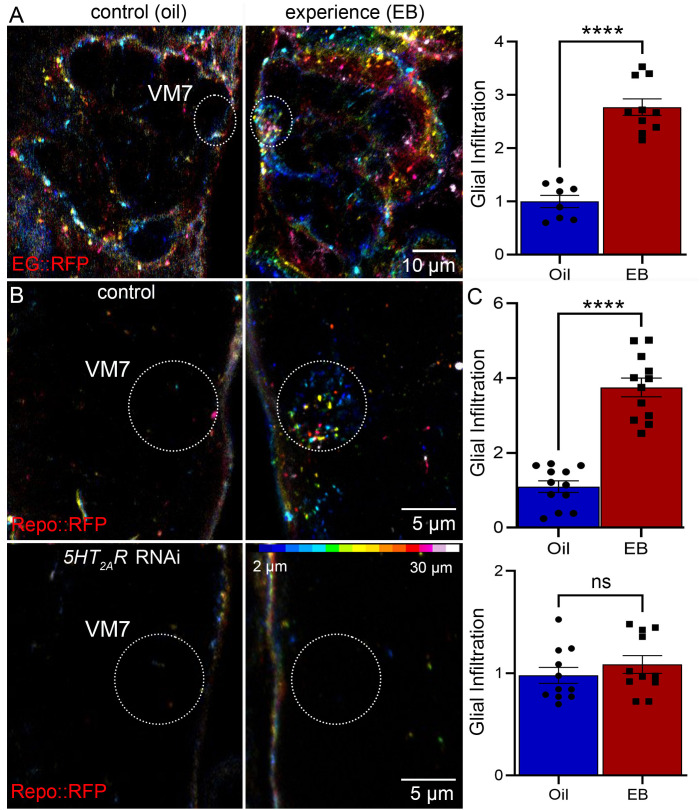
Disrupting glial 5-HT signaling prevents experience-dependent infiltration. **A**, Ensheathing glia *GMR56F03*-Gal4 driven UAS-MCD8::RFP displayed in 3D projection of the central brain antennal lobe in a color-coded Z-stack (scale bottom right in C), with 24-hour exposure to the mineral oil vehicle control (left) and EB odorant experience (right) from 0 to 1 days post-eclosion (dpe) in the critical period. The glia specifically infiltrate target VM7 glomeruli (dotted circles) with critical period experience. Right: Quantification of glial infiltration into VM7 normalized to the mineral oil control shows a significant increase of ensheathing glia with EB experience (*n* = 10/condition, *p* = 1.72 × 10^−7^). **B,** Testing the role of glial 5-HT_2A_R signaling in experience-dependent synaptic infiltration. Transgenic controls (top: *w*^*1118*^;* UAS-mCD8*::*RFP/+; repo-Gal4/mCD8-Or42a::GFP*) and glia-specific *5-HT*_*2A*_*R* RNAi (bottom: *w*^*1118*^;* UAS-mCD8*::*RFP/Or42a-mCD8*::*GFP; UAS-5-HT*_*2A*_*RNAi/repo-*Gal4) under the same conditions as above, at higher magnification. The control glial infiltration into target VM7 glomeruli (dotted circles) is completely blocked with glial *5-HT*_*2A*_*R* RNAi. **C**, Quantification of glial VM7 infiltration normalized to oil vehicle shows highly significant experience-dependent infiltration in controls (top: *n* = 12/condition, *p* = 7.00 × 10^−9^), but none with glial *5-HT*_*2A*_*R* RNAi (bottom: *n* = 11/condition, *p* = 0.370). Individual data points shown with mean ± SEM. Significance indicated as *p* < 0.0001 (****), *p* > 0.05 (not significant, ns). The data underlying this Figure can be found in S1 Data.

Glia are phagocytes that prune the synaptic glomeruli innervation [1,15]. In the *Drosophila* brain olfactory circuit, the ALG, EG, and CG have all been implicated as active phagocytes [51]. During critical period OSN synaptic pruning, EB experience specifically targets glial projections into the VM7 synaptic glomeruli neuropil [15]. Using glial class-specific Gal4 drivers (S1 Fig), we first investigated the experience-dependent infiltration from all of these glial classes (Fig 2). Newly eclosed juveniles at 0–1 days post-eclosion (dpe) were placed in vacuum-sealed odorant exposure chambers containing either the mineral oil vehicle alone (control) or EB odorant dissolved in the oil (experience). Critical period EB exposure drives experience-dependent glial infiltration VM7 synaptic innervation pruning via phagocytosis [1]. The glial projection infiltration can best be visualized as a 3-dimensional projection of the VM7 synaptic glomerulus (Fig 2). Different glial classes were labeled with a membrane-bound RFP driven by the class-specific Gal4 drivers (for example, EG::RFP) with the depth shown by color-coding at 0.75 μm intervals within the Z-stack confocal image (Fig 2). This 3-dimensional AL representation shows the relative position of glial cells in the control condition and following critical period EB experience. The EB-responsive target VM7 synaptic glomerulus is independently labeled via a direct fusion of the membrane-bound GFP directly to the Or42a promoter (Or42a-mCD8::GFP), with VM7 indicated as white dotted circles (Fig 2). For all quantified comparisons, glial projection infiltration into the VM7 synaptic glomeruli is normalized to the oil vehicle control condition.

With all 3 glial class-specific RFP membrane markers, glial infiltration into the VM7 synaptic glomerulus was assayed in the oil control and with EB experience. No detectable change in the distribution or number of ALG or CG occurs with EB exposure (S2 Fig), but there is a striking infiltration of the EG (Fig 2A). In oil vehicle controls, EG surround the AL neuropil with few or no detectable projections into synaptic glomeruli, specifically including VM7 (Fig 2A, left image; white dotted circle). Three-dimensional depiction of EG in the oil control indicates that glia remain associated with the AL boundary. In response to critical period EB experience, EG are the only class that infiltrate into the AL neuropil, and this movement is predominatly targeted to the EB-responsive VM7 synaptic glomerulus (Fig 2A, right image; white dotted circle). Other EG movements presumably reflect the presence of other EB-responsive glomeruli elsewhere in the AL. Quantification shows a very highly significant nearly 3-fold increase in EG VM7 infiltration following critical period EB experience compared to the oil vehicle control (Fig 2A, right). These results are consistent with the fact that EG mediate experience-dependent synaptic innervation pruning during the critical period [15]. We next turned to investigating the guidance signaling that specifically targets EG into the EB-responsive VM7 synaptic glomerulus in this critical period mechanism.

Serotonin (5-HT) intercellular signaling is well established to shape olfactory circuit connectivity [52,53], and our previous work found glial 5-HT_2A_ receptors upregulated in VM7 glomeruli in response to critical period EB experience [21]. To test this mechanism in EG infiltration, we used a glial driver (*repo*-Gal4) to express *5-HT*_*2A*_*R* RNAi while simultaneously labeling glia with an RFP membrane marker. In the transgenic driver controls with the oil vehicle, there is again a complete absence of any glia within the VM7 synaptic glomerulus (Fig 2B, top left image; white dotted circle). With critical period EB experience, glial projections again strongly and specifically infiltrate the VM7 glomerulus (Fig 2B, top right image; white dotted circle). Quantification shows a >3-fold increase in glial infiltration in response to the EB experience compared to oil vehicle control (Fig 2C, top). With glial *5-HT*_*2A*_*R* RNAi, there is a total loss of infiltration targeted by EB experience. In the oil vehicle control, there is no glial infiltration and, in sharp contrast to the above genetic control, EB experience results in no VM7 infiltration, making it indistinguishable from the control condition (Fig 2B, bottom row). Quantification shows there is absolutely no change in VM7 glial infiltration between the oil vehicle control and EB experience conditions (Fig 2C, bottom). Additional controls confirm blocked infiltration is a direct result of 5HT_2A_R loss and not the presence of a second UAS genetic construct (S3 Fig). Thus, glial *5-HT*_*2A*_*R* RNAi blocks experience-dependent glial infiltration into VM7 synaptic glomeruli. We next tested glial class-specific roles in experience-dependent synaptic pruning during this juvenile critical period.

### Ensheathing glia serotonin signaling drives experience-dependent synapse pruning

**Fig 3:**
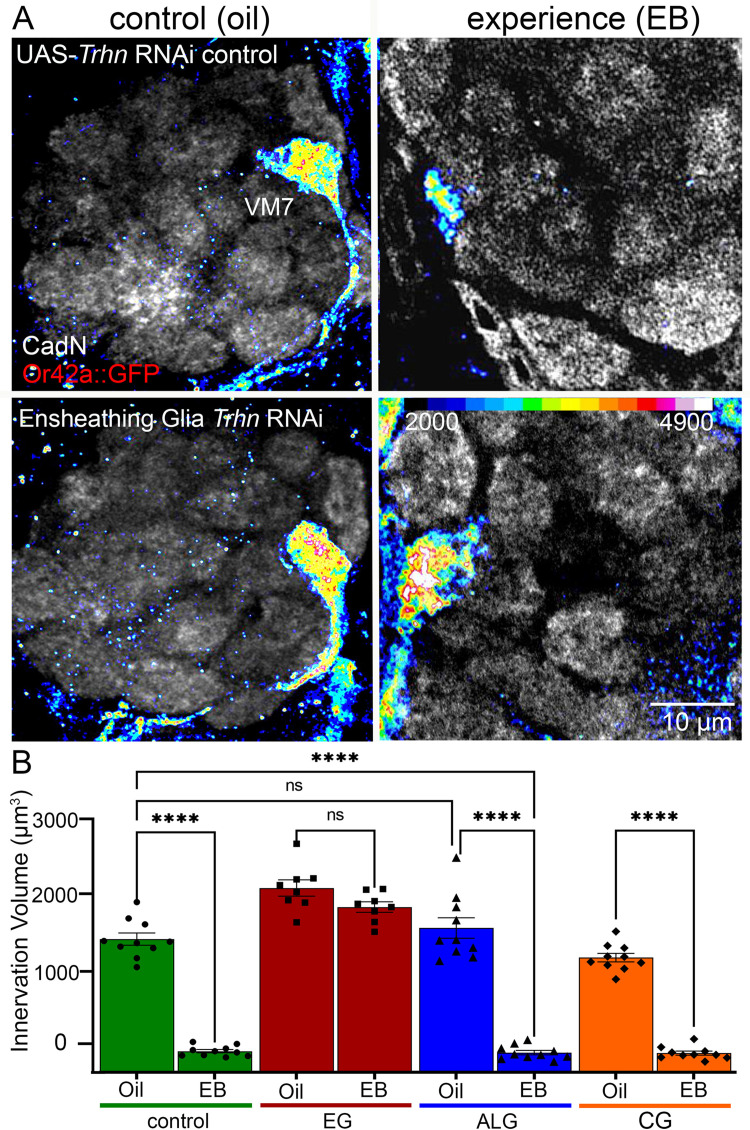
Ensheathing glia produce serotonin for critical period synapse pruning. **A,** Or42a neuron innervation of VM7 synaptic glomeruli shown with Or42a::GFP labeling (colored heat-map,16 LU scale; bottom right), co-labeled with anti-N-Cadherin (CadN) for visualization of all synaptic glomeruli (gray scale). The top row shows transgenic control of UAS-*Trhn* RNAi alone (*w*^*1118*^;* Or42a-mCD8*::*GFP/+; UAS-Trhn RNAi/+*) with oil vehicle (left) or EB odorant experience (right) for 24 hours from 0 to 1 dpe in the critical period. The bottom row shows ensheathing glia *GMR56F03*-Gal4 driven *Trhn* RNAi (*w*^*1118*^;* Or42a-mCD8*::*GFP/+; UAS-Trhn RNAi/GMR56F03*-Gal4) with experience-dependent synaptic glomeruli pruning completely blocked. **B**, Quantification of Or42a OSN innervation volume in the *Trhn* RNAi control (green, left), and driven in ensheathing glia (EG, red, second), astrocyte-like glia (ALG, blue, third; *w*^*1118*^;* GMR86E01*-Gal4; *UAS-Trhn RNAi*), and cortex glia (CG, orange, right; *w*^*1118*^;* GMR54H02*-Gal4; *UAS-Trhn RNAi*). Two-way ANOVA with Tukey’s multiple comparison shows glial pruning of Or42a OSN innervation in the *Trhn* RNAi control (*n* = 10/condition, *p* < 1.00 × 10^−15^), as well as with astrocyte-like glia and cortex glia *Trhn* RNAi (ALG: *n* = 10/condition, *p* < 1.0 × 10^−15^; CG: *n* = 10/condition, *p* < 1.0 × 10^−15^), but no significant pruning with ensheathing glia *Trhn* RNAi (*EG*: *n* = 8, *p* = 0.379). All data points shown with mean ± SEM. Significance: *p* < 0.0001 (****) and *p* > 0.05 (ns). The data underlying this Figure can be found in S1 Data.

Tryptophan hydroxylase (Trhn) is an essential enzyme for serotonin synthesis from the tryptophan precursor, and cell-targeted *Trhn* RNAi can be used to test serotonergic signaling requirements [54]. Our previous work discovered that Trhn is required in glia, and not at all in neurons, for critical period EB experience-dependent synapse pruning [21]. We hypothesized that ALG, which are not mediating synaptic pruning but are implicated in serotonergic mediation during circuit remodeling [26,55], likely use serotonin as a core regulatory signal in response to experience during the critical period. To test glial class-specific requirements, we used an EG-specific driver (*GMR56F03-*Gal4), an ALG-specific driver (*GMR86E01-*Gal4), and a CG-specific driver (*GMR54H02*-Gal4) to express UAS-*Trhn* RNAi in each class. Each Gal4 demonstrates specificity to each of the glial class (S1 Fig) and similar expression strength without genetic dilution effects (S3 and S4 Figs). In the UAS-*Trhn* RNAi control (no Gal4 driver present) and all 3 glial class knockdowns, we paired 24-hour critical period exposure (0–1 dpe) of the oil vehicle (control) to EB odorant dissolved in oil (experience). We then tested experience-dependent pruning of Or42a OSN innervation in the VM7 glomerulus, with all synaptic glomeruli labeled with an N-Cadherin antibody (CadN; gray scale), and VM7 innervation labeled by Or42a receptor-direct fusion to the membrane mCD8::GFP (Or42a::GFP; shown in an intensity heat-map, see color scale). Representative images and quantification of the 3-dimensional Or42a neuron innervation volume in the VM7 synaptic glomeruli are shown in Fig 3.

In the UAS-*Trhn* RNAi/+ control, 24-hour EB experience from 0 to 1 dpe in the critical period causes strong VM7 synaptic glomeruli pruning compared to the oil vehicle control (Fig 3A, top row). The heat-map label shows the pruned Or42a OSN innervation (color) with all synaptic glomeruli marked by anti-CadN (gray scale). In sharp contrast, blocking serotonin synthesis in only EG fully prevents experience-dependent pruning, with the EB experience indistinguishable from oil vehicle controls (Fig 3A, bottom row). UAS-*Trhn* RNAi driven in either ALG or CG allows pruning identical to control, with both conditions showing the same VM7 innervation loss in response to EB experience. Quantification of the innervation volumes reveals highly significant pruning in transgenic controls (green), and with ALG (blue) and CG (orange) *Trhn* RNAi from EB experience compared to the oil vehicle (Fig 3B). The control (green) is a 6.3-fold volumetric decrease, ALG (blue) is a 7.2-fold volumetric decrease, and CG (orange) is a 6.0-fold volumetric decrease in the VM7 innervation. In contrast, EG *Trhn* RNAi results in no experience-dependent pruning (Fig 3B, red). Quantification reveals no significant innervation volume change. These findings reveal that only the EG require serotonin synthesis via tryptophan hydroxylase for serotonergic signaling to drive critical period experience-dependent synapse pruning, an acute requirement that is independent of *Trhn* loss during earlier development (S5 Fig). We next tested glial class(es) requiring the 5-HT_2A_ receptor for this signaling mechanism during the early-life pruning mechanism in the juvenile brain.

### Astrocyte-like 5-HT_2A_ receptor function is essential for critical period synaptic pruning

To test specific requirements across different glial classes for serotonin signaling, we moved forward to the reception side of this serotonergic communication. The 5-HT_2A_ G-protein-coupled receptor is well established to be a central regulator of brain circuit plasticity [23,24], and our previous work discovered a glial 5-HT_2A_ receptor requirement in olfactory critical period experience-dependent synaptic pruning [21]. Based on the above discovery of the serotonin signaling role in EG, we hypothesized that a distinct glial class should be required for the signaling reception, with 5-HT_2A_ receptors mediating a novel mechanism of glial-to-glia class signaling. 5-HT_2A_ receptors are present in different glia subclasses and have distinct roles in circuit maintenance and synaptic regulation, including roles in mammalian astrocytes and microglia [56–58]. The different glia classes performing separable tasks in experience-dependent synaptic remodeling suggests communication not only with neurons, but also between different glial classes. We therefore hypothesized glia-to-glia class signaling via the 5-HT_2A_ receptor is required for olfactory experience-dependent synaptic pruning. To test this hypothesis, we again used EG-specific driver (*GMR56F03-*Gal4), ALG-specific driver (*GMR86E01-*Gal4), and CG-specific driver (*GMR54H02*-Gal4) to express UAS-*5-HT*_*2A*_*R RNAi* and then assayed Or42a neuron innervation pruning in the VM7 synaptic glomeruli in response to EB experience compared to the oil vehicle during the 0–1 dpe critical period.

**Fig 4:**
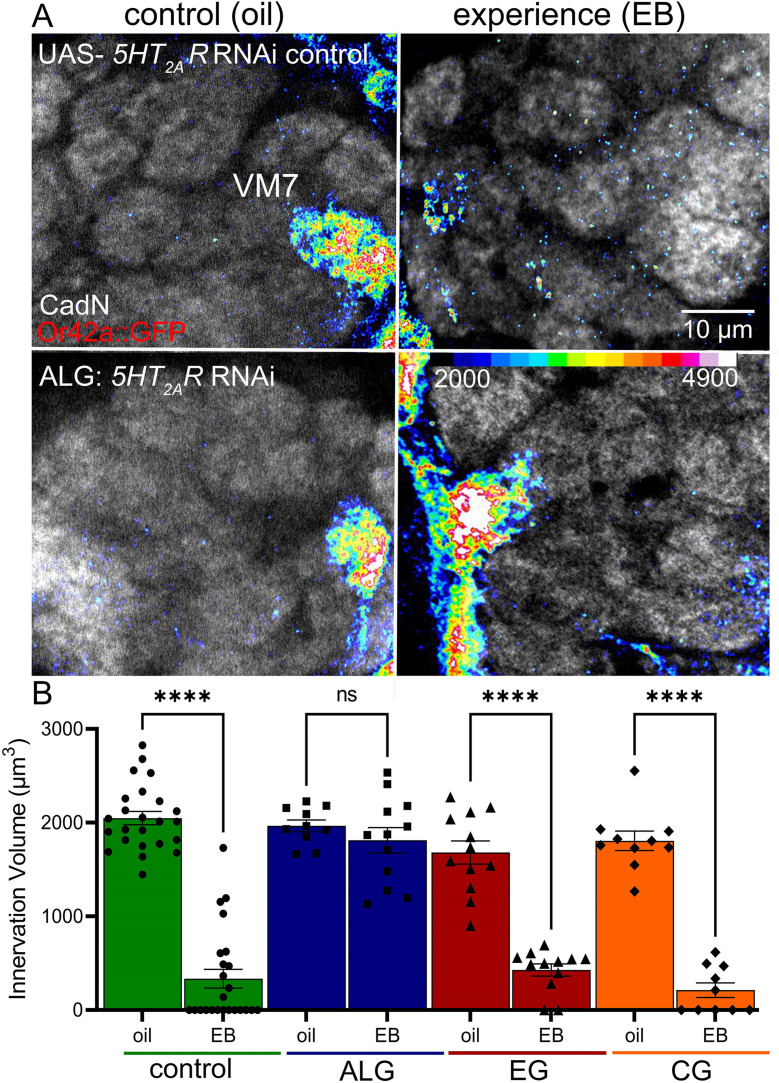
Astrocyte-like glia 5-HT_2A_R is required for experience-dependent pruning. **A,** Or42a OSN innervation of VM7 glomeruli (Or42a::GFP) shown as a colored heat-map (16 LU scale, bottom right) with anti-CadN labeling of all synaptic glomeruli (gray scale). Top row: *5-HT*_*2A*_*R* RNAi alone transgenic control (*w*^*1118*^;* Or42a-mCD8*::*GFP/+; UAS-5-HT*_*2A*_*R RNAi/+*) with oil vehicle (left) or EB experience (right) for 24 hours from 0 to 1 dpe. Bottom row: astrocyte-like glia (ALG) *GMR86E01*-Gal4 *5-HT*_*2A*_*R RNAi* (*w^1118^; Or42a-mCD8::GFP/+; UAS-5-HT_2A_R RNAi/GMR86E01*-Gal4) showing experience-dependent synaptic glomeruli pruning completely blocked. **B**, Quantification of VM7 innervation volume in the *5-HT_2A_R* RNAi alone control (green, left), and driven in ALG (red, second), ensheathing glia (EG, blue; *w^1118^; Or42a-Gal4/+; UAS-5-HT_2A_R RNAi/GMR56F03*-Gal4), and cortex glia (CG, orange; *w*^*1118*^;* Or42a-Gal4/+; UAS-5-HT*_*2A*_*R RNAi/GMR54H02*-Gal4). Two-way ANOVA with Tukey’s multiple comparison shows significant glial pruning in the *5-HT*_*2A*_*R* RNAi control (*n* = 24/condition, *p* = 5.00 × 10^−14^), as well as ensheathing and cortex glia *5-HT*_*2A*_*R* RNAi conditions (EG: *n* = 10/ condition, *p* = 2.55 × 10^−11^; CG: *n* = 10/condition, *p* = 1.58 × 10^−13^), but no significant pruning with astrocyte-like glia *5-HT*_*2A*_*R* RNAi (*ALG*: *n* = 10, *p* = 0.980). All individual data points are shown with the mean ± SEM. Significance is indicated as *p* < 0.0001 (****) and *p* > 0.05 (not significant, ns). The data underlying this Figure can be found in S1 Data.

In the UAS-*5-HT*_*2A*_*R* RNAi/+ controls, 24-hour EB experience causes the expected VM7 synaptic glomeruli pruning compared to the oil vehicle control (Fig 4A, top row). The heat-map shows Or42a OSN innervation pruning (color) with synaptic glomeruli labeled by anti-CadN (gray scale). Blocking 5-HT_2A_ receptor signaling in only ALG prevents experience-dependent pruning (Fig 4A, bottom row). Conversely, *5-HT*_*2A*_*R* RNAi in either EG or CG has no effect, with both showing the same loss of VM7 innervation in response to critical period EB experience. Quantification of innervation volume reveals highly significant experience-dependent pruning in the RNAi control (green) and with *5-HT*_*2A*_*R* RNAi driven in EG (blue) or CG (orange; Fig 4B). The control (green) shows a 6-fold decrease, EG (blue) a nearly 5-fold decrease, and CG (orange) a >7-fold decrease in VM7 innervation volume. In contrast, the ALG *5-HT*_*2A*_*R* RNAi results in a complete block of synaptic glomerulus pruning (Fig 4B, red). Quantification reveals no significant experience-dependent innervation volume change. These findings indicate the 5-HT_2A_ receptor is required only in ALG for critical period synaptic pruning. In combination with the above *Trhn* results showing EG serotonin signaling, we suggest that a communication mechanism from EG to ALG is essential for experience-dependent synaptic pruning. We next tested if 5-HT_2A_Rs in ALG only drives this critical period signaling mechanism.

### Restoring 5-HT_2A_R function only in astrocytes rescues 5-HT_2A_R null synapse pruning

**Fig 5:**
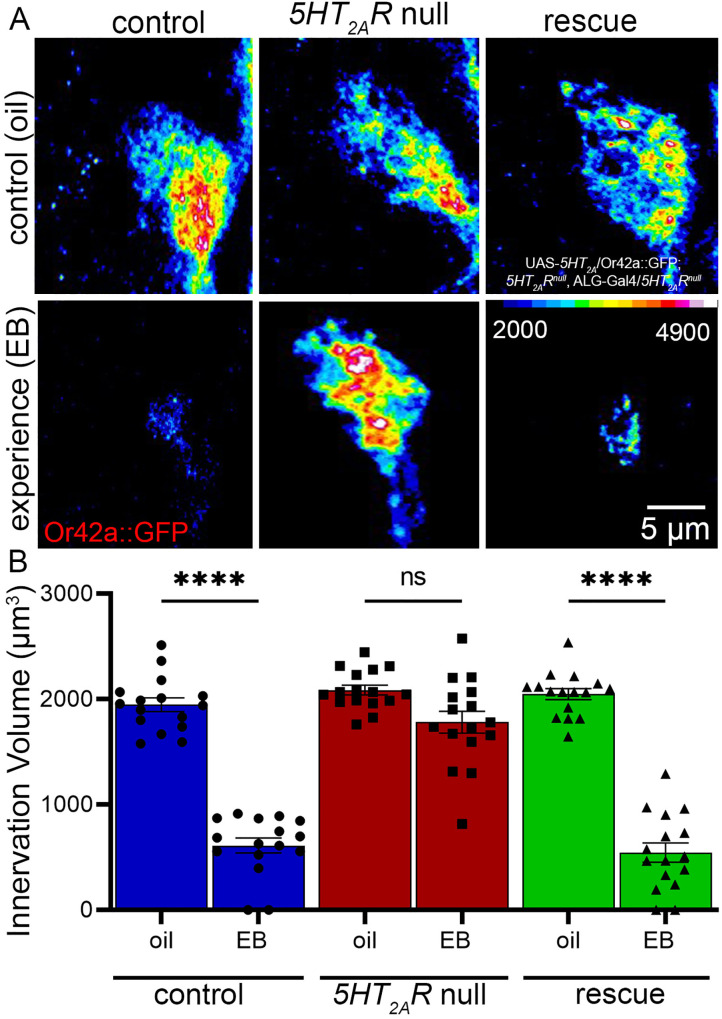
Restoring astrocyte-like glia 5-HT_2A_R rescues pruning in a *5-HT*_*2A*_*R* null. **A**, Or42a OSN innervation of VM7 glomeruli (Or42a::GFP) shown as a colored heat-map (16 LU scale, bottom right). Top row: oil vehicle treatment of the transgenic control (left; *w*^*1118*^;* Or42a-mCD8*::*GFP/+; GMR86E01-Gal4/+), the 5-HT_2A_R* null mutant (center, *w*^*1118*^;* Or42a-mCD8*::*GFP/+; 5-HT*_*2A*_*R*^*null*^*/5-HT*_*2A*_*R*^*null*^), and ALG-targeted rescue condition (right, *w*^*1118*^;* Or42a-mCD8*::*GFP/UAS-5-HT*_*2A*_*R; 5-HT*_*2A*_*R/5-HT*_*2A*_*R, GMR86E01*-Gal4*/5-HT_2A_R*). Bottom row: The critical period (0−1 dpe) EB exposure to the same genotypes. Restoring 5-HT_2A_ receptor in astrocyte-like glia (bottom right) rescues the blocked pruning in the null (bottom center). **B**, Quantification of Or42a OSN synaptic glomeruli innervation volumes for the control (blue, left), *5-HT_2A_R* null (red, center), and ALG-targeted rescue condition (green, right) with oil vehicle and EB experience treatments. Compared to experience-dependent synaptic glomeruli pruning in the controls (*n* = 16/condition, *p* = 4.36 × 10^−10^), the *5-HT*_*2A*_*R* null blocks pruning (*n* = 16/condition, *p* = 0.053), which is restored by ALG-targeted *5-HT*_*2A*_*R* rescue (*n* = 16/condition, *p* = 4.35 × 10^−10^). All individual data points are shown with mean ± SEM. Significance is indicated as *p* < 0.0001 (****) and *p* > 0.05 (not significant, ns). The data underlying this Figure can be found in S1 Data.

One of the best compliments to test cell-type-specific signaling is targeted genetic rescue of a global null mutant [59]. This approach tests the sufficiency of the signaling mechanism. The *Drosophila* genetic toolkit provides a robust system to pursue rescue experiments for cellular signaling requirements [60]. To understand the requirements of the 5-HT_2A_ receptor in the ALG, we needed to test if this glial class alone requires the receptor to mediate experience-dependent synapse pruning during the juvenile critical period. Our previous work discovered a rate-limiting requirement of glial 5-HT_2A_ receptors using both targeted knockdown and overexpression [21]. We also found above that the 5-HT_2A_R requirement is within only the ALG, and that 5-HT_2A_ receptor knockdown in EG and CG does not affect experience-dependent synapse pruning. We therefore hypothesized that the 5-HT_2A_ receptor in the ALG is sufficient to enable this critical period mechanism. To test this hypothesis, we performed cell-targeted rescue in ALG in a global 5-HT_2A_ receptor null mutant generated via CRISPR/Cas9 gene editing [61]. We employed the ALG *GMR86E01-*Gal4 to drive wildtype UAS-*5-HT*_*2A*_*R* in this *5-HT*_*2A*_*R* null background. We used the same oil vehicle and EB experience conditions as above to analyze experience-dependent pruning within the defined critical period. Representative images and quantification of 3-dimensional Or42a neuron innervation volume in the VM7 synaptic glomeruli are shown in Fig 5.

In the transgenic controls, we find the expected levels of experience-dependent synaptic glomerulus pruning compared to the oil vehicle control (Fig 5A, left) and a block of pruning in the *5-HT*_*2A*_ *receptor* global null mutants (Fig 5A, center). The Or42a::GFP fusion heat-map visualization shows the VM7 innervation with and without glial pruning. In the ALG 5-HT_2A_ receptor rescue condition, we find that expression of the 5-HT_2A_R in only this single glial class is sufficient to induce experience-dependent pruning in otherwise *5-HT*_*2A*_*R* null mutants (Fig 5A, right). Innervation quantification of transgenic controls shows highly significant experience-dependent synaptic glomerulus pruning, with a 4-fold volume loss compared to the oil vehicle (Fig 5B, blue). In contrast, the *5-HT*_*2A*_*R* global null (Or42a-mCD8::GFP/+; *5-HT*_*2A*_*R*^*nu*ll^) shows no significant change in innervation volume in response to critical period EB experience (Fig 5B, red). In the ALG-targeted rescue (UAS-*5-HT*_*2A*_*R*/Or42a-mCD8::GFP; *GMR86E01*-Gal4, *5-HT*_*2A*_*R*^*null*^*/5-HT*_*2A*_*R*^*null*^), a highly significant degree of EB experience-dependent synaptic glomerulus pruning is indistinguishable from control, with again a 4-fold volume loss compared to the oil vehicle (Fig 5B, green). This result shows that 5-HT_2A_ receptors in the ALG is necessary and sufficient for synaptic pruning in response to olfactory experience in the juvenile critical period. We next explored the scope of ALG 5-HT_2A_ receptor signaling requirement by testing if conditional adult expression is sufficient to re-open “critical period-like” plasticity at maturity.

### Adult astrocyte 5-HT_2A_R expression re-opens experience-dependent synapse remodeling

**Fig 6:**
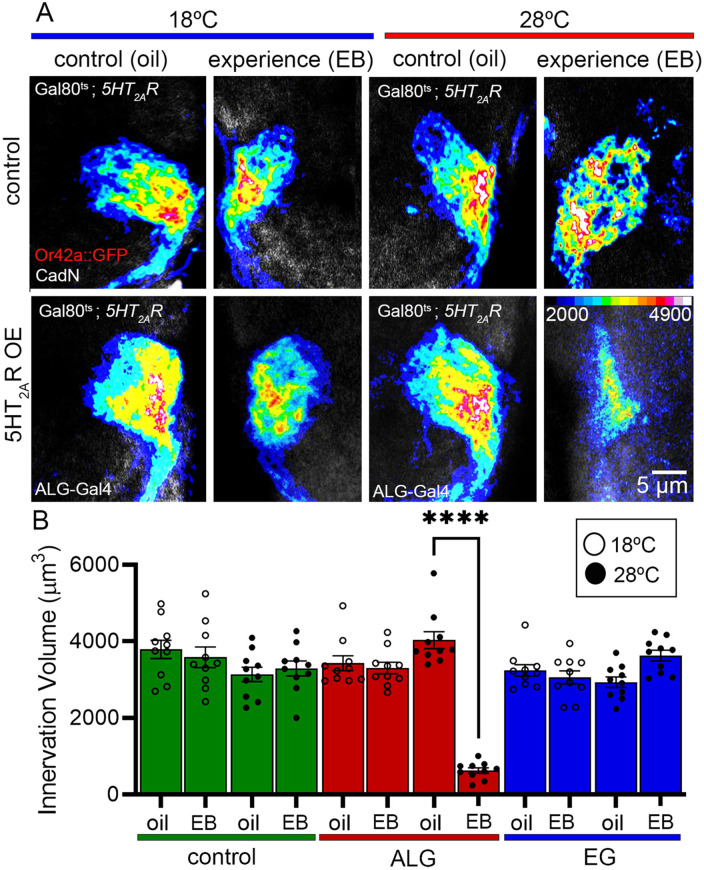
Conditional adult astrocyte *5-HT*_*2A*_R expression induces synaptic pruning. **A**, Mature adults exhibit no experience-dependent pruning of the Or42a OSN innervation. Left (18°C, blue, Gal80^ts^ permissive temperature): Genetic background control (top: *w*^*1118*^;* tubulin-Gal80*^*ts*^*/UAS-5-HT*_*2A*_*R; Or42a-mCD8*::*GFP/+*) and with ALG-specific Gal4 driver (bottom: *w*^*1118*^;* tubulin-Gal80*^*ts*^*/UAS-5-HT*_*2A*_*R; Or42a-mCD8*:*GFP/ALG; GMR86E01-Gal4*) with 24-hour oil vehicle (left) or EB experience (right). There is no experience-dependent pruning with Gal80^ts^ active. Right (28°C, red, Gal80^ts^ restrictive temperature): The same genotypes and conditions with Gal80^ts^ repressed in the lower row to enable ALG-specific Gal4 driver conditional 5-HT_2A_R expression in the control oil vehicle (lower left) and with EB experience (lower right). Experience-dependent synaptic glomerulus pruning induced in mature adults. **B,** Quantification of Or42a OSN innervation volumes at permissive 18°C (white circles) and restrictive 28°C (black circles) for genetic background controls (green), ALG-specific 5-HT_2A_R (red), and ensheathing glia (EG) 5-HT_2A_R (blue; *w*^*1118*^;* tubulin-Gal80*^*ts*^*/UAS-5-HT*_*2A*_*R; Or42a-mCD8*:*GFP/GMR56F03*-Gal4). Tests by two-way ANOVA with Tukey’s multiple comparison tests show no significant change in innervation volumes in background controls (green, left; *n* = 10/condition, 18°C *p* = 0.626, 28°C *p* = 0.999), EG conditions (blue, right; *n* = 10/condition, 18°C *p* = 0.999, 28°C *p* = 0.2673), or ALG at 18°C (red, left; *n* = 10/condition, 18°C *p* = 0.999). Only ALG-specific 5-HT_2A_R at 28°C causes pruning with EB experience at maturity (red, right; *n* = 10/condition, 28°C *p* = 1.0 × 10^−15^). All data points are shown with the mean ± SEM. Significance is indicated as *p* < 0.0001 (****). The data underlying this Figure can be found in S1 Data.

The objective of “re-opening” the critical period to harness remodeling capacities is the aspiration for a wide spectrum of serotonergic therapeutic strategies [28,62], given connectivity defects causing life-long and debilitating impairments [63]. Our previous work discovered glial-targeted 5-HT_2A_ receptor expression in mature adults enables de novo experience-dependent synaptic pruning [21]. We hypothesized ALG 5-HT_2A_ receptor function mediates this mechanism. To test this idea, we employed a conditional, temperature-sensitive Gal80 (Gal80^ts^) transcriptional repressor to express the 5-HT_2A_ receptor in only adult astrocytes. Gal80^ts^ blocks Gal4-driven transcription at permissive temperature (18°C), but inactivates at restrictive temperature (28°C) to permit Gal4 transcription [64]. Control adults at permissive and restrictive temperatures show no EB experience-dependent pruning of VM7 synaptic innervation (Fig 6A, top). At permissive temperature (18°C), when Gal80^ts^ represses 5-HT_2A_ receptor expression in the ALG, there is likewise no detectable experience-dependent pruning (Fig 6A, bottom). Similarly, at restrictive temperature (28°C) when Gal4 is no longer repressed by Gal80^ts^, the controls show no synaptic glomeruli pruning with EB experience compared to controls with no 5-HT_2A_ receptor expression (Fig 6A, top right). Quantified innervation volumes in all 3 control groups confirm that there is no significant pruning of the VM7 synaptic innervation in mature adults at either temperature (18°C, 28°C) with the absence of the 5-HT_2A_ R expression in ALG (Fig 6B, green and red).

Conditional ALG 5-HT_2A_ receptor expression only in mature adults induces experience-dependent synaptic glomeruli pruning, which appears identical to the juvenile critical period mechanism (Fig 6A, bottom right). At the restrictive temperature (28°C), controls that lack the Gal4 driver have densely innervated VM7 synaptic glomeruli in both the oil vehicle and EB odorant experience conditions, showing a complete lack of experience-dependent synaptic glomeruli pruning (Fig 6A, right top row). In these exact same conditions, but with Gal4 now driving 5-HT_2A_ receptor expression only in the adult ALG, there is strong experience-driven synaptic glomeruli pruning consistent with the juvenile critical period mechanisms (Fig 6A, right bottom row). Quantification of the VM7 innervation volumes for all conditions are shown in Fig 6B. At the permissive temperature (18°C, open circles), when functional Gal80^ts^ is actively repressing 5-HT_2A_ receptor expression, there is a complete lack of experience-dependent synaptic glomeruli pruning in comparison to the oil vehicle controls (Fig 6B). At the restrictive temperature (28°C, black circles), when the Gal80^ts^ is inactive, the ALG 5-HT_2A_ receptor expression drives very highly significant experience-dependent pruning in mature adults (Fig 6B, red). Quantification shows >4-fold innervation volume loss in the VM7 synaptic glomeruli at the restrictive 28°C at adult maturity in response to experience. In contrast, EG 5-HT_2A_R expression causes no detectable experience-dependent innervation pruning in the VM7 synaptic glomeruli (Fig 6B, blue). Thus, ALG 5-HT_2A_R expression alone re-opens “critical period-like” capabilities. We next turned to exploring the mechanism of the 5-HT_2A_R signaling requirement.

### MMP-1 is induced by experience and required for experience-dependent synaptic pruning

**Fig 7:**
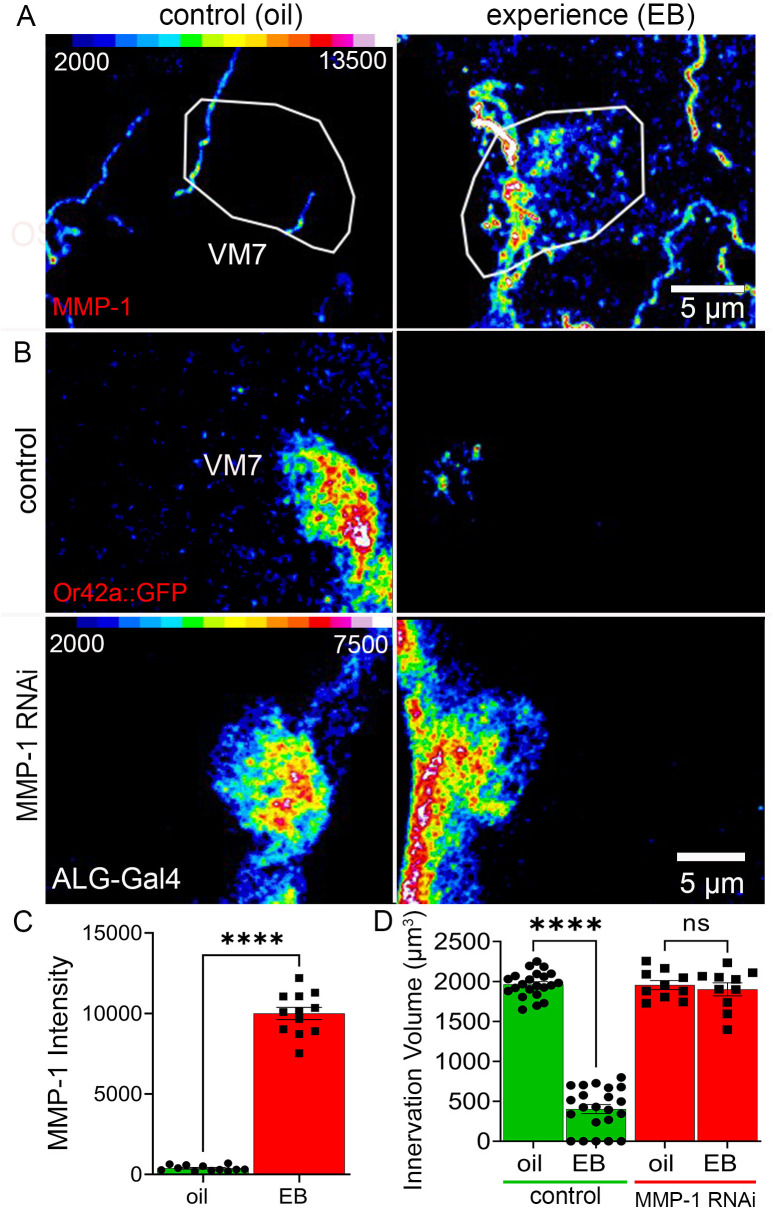
Experience-induced MMP-1 required for experience-dependent pruning. **A**, Anti-MMP1 labeling shown as a color-coded Z-stack (scale, top left) in VM7 glomeruli (white ovals), with 24-hour exposure from 0 to 1 dpe to the mineral oil vehicle control (left) or 25% EB odorant (right). **B**, Astrocyte-like glia control (top: *w*^*1118*^;* Or42a-mCD8::GFP/+; GMR86E01-*Gal4/+) and *MMP-1* RNAi targeted to the astrocyte-like glia (bottom: *w*^*1118*^;* Or42a-mCD8::GFP/+; GMR86E01-*Gal-4 RNAi/UAS-Dicer-2) in color-coded Z-stack of Or42a::GFP labeling of the VM7 innervation (scale, bottom left) with 24-hour exposure from 0 to 1 dpe to the mineral oil vehicle control (left) or 25% EB odorant (right). **C**, Quantification of the anti- MMP-1 fluorescence intensity (*n* = 12/condition, *p*-value ≤ 1.0 × 10^−15^). The individual data points are shown with the mean ± SEM. Significance indicated as *p* < 0.0001 (****). **D**, Quantification of the Or42a OSN innervation volume in the ALG-Gal4 transgenic driver control (green) and with ALG-targeted *MMP-1* RNAi (red). Two-way ANOVA with Tukey multiple comparison shows significant experience-dependent pruning of VM7 innervation in the control (green, *n* = 22/condition, *p*-value = 2.0 × 10^−11^), whereas pruning is completely blocked by ALG-targeted *MMP-1* RNAi (red, *n* = 10/condition, *p*-value = 0.950). Individual data points shown with mean ± SEM. Significance: *p* < 0.0001 (****) and *p* > 0.05 (ns). The data underlying this Figure can be found in S1 Data.

5-HT_2A_R signaling regulates ECM turnover controlling synapse stability [37], which suggests the mechanistic role in ALG during critical period synapse pruning. Specifically, 5-HT_2A_R signaling activates MMP production for ECM remodeling [39]. Compared to the ~25 MMPs within mammals, *Drosophila* has a reduced complement of only two MMPs; MMP-1 and MMP-2. These MMPs are implicated in diverse neuronal cellular remodeling mechanisms [32], but MMP-1 activation in glia has been specifically shown to mediate glial pruning of neuronal axons [41]. We therefore focused on MMP-1 as the likely candidate for experience-dependent 5-HT_2A_R signaling. We hypothesized that glial MMP-1 would be upregulated in response to critical period EB experience, and that this MMP-1 induction would be required for experience-dependent synaptic glomerulus pruning. To test this hypothesis, we first exposed juveniles during the early-life critical period (0–1 dpe) to either the mineral oil vehicle control or EB odorant and employed MMP-1 immunocytochemical labeling to visualize protein dynamics during experience-dependent innervation pruning. The oil vehicle controls show very low basal levels of MMP-1 in the VM7 glomeruli (Fig 7A, left). In sharp contrast, EB experience dramatically elevates MMP-1 expression (Fig 7A, right). Quantifications of MMP-1 levels within the VM7 synaptic glomerulus show critical period experience causes a highly significant increase (>25-fold) compared to matched controls (Fig 7C). Thus, MMP-1 is induced in glia by EB experience during the critical period.

We hypothesized MMP-1 would be required in astrocyte glia specifically to enable experience-dependent synaptic glomerulus pruning. To investigate this requirement, we employed ALG-Gal4 *MMP-1* RNAi together with Or42a::GFP visualization of the VM7 innervation (Fig 7B). We exposed the transgenic ALG-Gal4 driver control and targeted *MMP-1* knockdown to either mineral oil vehicle control or EB odorant for 24 hours from 0 to 1 dpe during the critical period. The controls show the characteristic innervation in the oil vehicle and the robust synaptic glomerulus pruning in response to EB experience, as expected (Fig 7B, top). ALG-Gal4 *MMP-1* RNAi show a block of experience-dependent synaptic glomerulus pruning, with no indication of decreased VM7 innervation (Fig 7B, bottom). Thus, *MMP-1* knockdown specifically in the ALG strongly prevents glial pruning in response to critical period experience. Specificity was further confirmed by *MMP-1* knockdown in EG, which failed to effect experience-dependent glial infiltration or VM7 innervation pruning (S6 Fig). Quantification of VM7 innervation volume reveals the expected EB experience-dependent extensive pruning and loss of innervation in transgenic driver controls (>3-fold innervation volume decrease compared to the vehicle; Fig 7D, left, green). In sharp contrast, critical period EB experience results in no detectable pruning with ALG *MMP-1* knockdown, with no significant VM7 innervation volume change compared to the vehicle (*p* = 0.950; Fig 7D, right, red). Comparable experiments investigating MMP-2 reveal no role in this mechanism. MMP-2 knockdown in glia permits pruning indistinguishable from control (S7A Fig), with MMP-1 loss alone specifically disrupting critical period synaptic glomerulus pruning (S7B Fig). Thus, critical period experience induces MMP-1 expression in VM7-infiltrating ALG that is required for experience-dependent synaptic glomerulus pruning.

### 5-HT_2A_R knockdown with MMP-1 overexpression restores experience-dependent pruning

**Fig 8:**
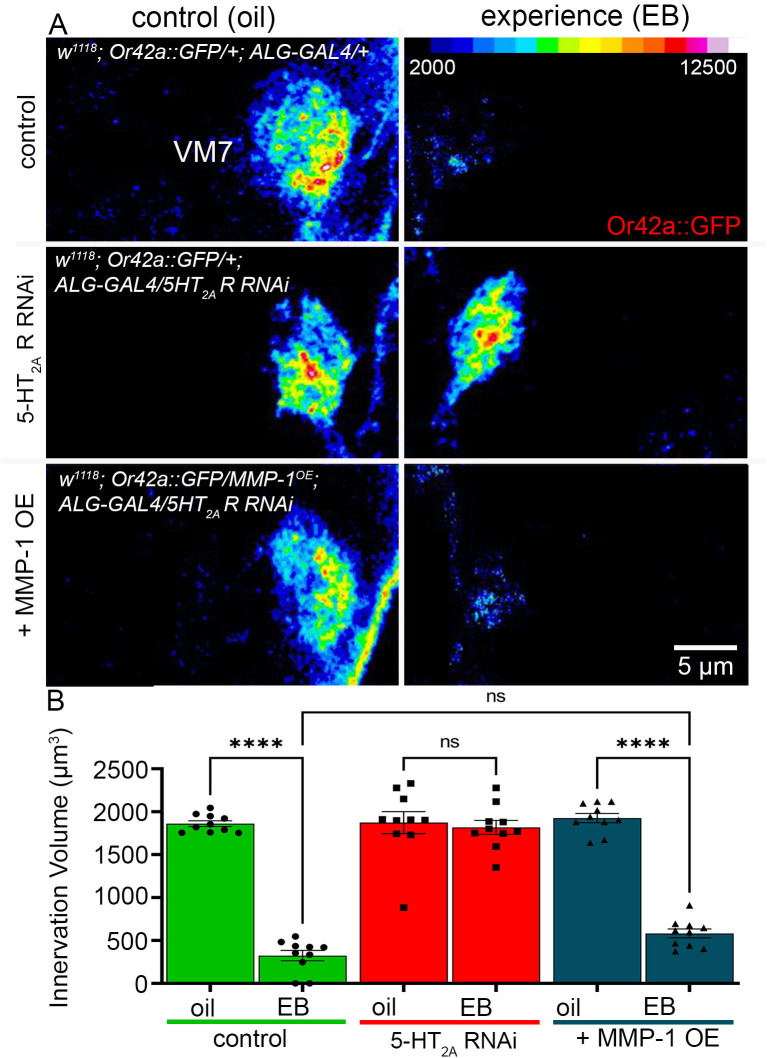
*MMP-1* OE restores *5HT*_*2A*_*R*-deficient experience-dependent pruning. **A**, Or42a OSN innervation of VM7 glomeruli shown as a color-coded Z-stack (scale, top right) with 24-hour exposure from 0 to 1 dpe to the mineral oil vehicle control (left) and EB odorant (right) in astrocyte-like glia control (top: *w*^*1118*^;* Or42a-mCD8::GFP/+; GMR86E01-Gal4/+*), the astrocyte-like glia *5-HT*_*2A*_*R* RNAi alone (middle: *w*^*1118*^;*Or42a-mCD8::GFP/+; GMR86E01-Gal4/UAS-5-HT*_*2A*_*R RNAi*), and with *MMP-1* overexpression (bottom: *w*^*1118*^;*Or42a-mCD8::GFP/UAS-MMP1; GMR86E01-Gal4/UAS-5-HT*_*2A*_*R RNAi*). **B**, Quantified Or42a OSN innervation volume in the transgenic control (green), *5-HT*_*2A*_*R* RNAi (red), and together with *MMP-1* overexpression (OE, blue). Two-way ANOVA with Tukey’s multiple comparison shows consistent significant innervation pruning in the transgenic control (green, *n* = 10/condition, *p*-value = 4.38 × 10^−13^). Targeted *5-HT*_*2A*_*R* RNAi results in no significant difference between oil control and EB experience conditions (red, *n* = 10/condition, *p*-value = 0.990). *MMP-1* OE restores synaptic pruning with a significant decrease of innervation volume in response to the EB odorant experience (blue, *n* = 10/condition, *p*-value = 4.43 × 10^−13^), which is nearly identical to the control. Individual data points are shown with mean ± SEM. Significance: *p* < 0.0001 (****) and *p* > 0.05 (ns). The data underlying this Figure can be found in S1 Data.

Our final objective was to test whether ALG 5-HT_2A_R signaling acts via MMP-1 induction as the mechanism for experience-dependent synaptic glomerulus pruning. We hypothesized this signaling cascade would enable glial infiltration into the VM7 synaptic neuropil, since it is known that this mechanism MMP remodeling of the synaptic ECM [38]. Serotonergic 5-HT_2A_R signaling triggers MMP extracellular proteolytic enzyme functions in other context [39], so we hypothesized the same mechanism here. To test the direct connection of experience-driven glial 5-HT_2A_R signaling to activating MMP-1 function, we employed MMP-1 overexpression with 5-HT_2A_R RNAi knockdown in the same ALG. This test determines whether MMP-1 overexpression in the absence of 5-HT_2A_R signaling is sufficient for experience-dependent synaptic glomerulus pruning. Experience was tested with 24-hour (0–1 dpe) exposure to EB odorant compared to oil vehicle control in 3 matched genetic groups: (1) the transgenic driver control (*w*^*1118*^;* Or42a-mCD8::GFP/+; GMR86E01-*Gal4 (ALG)/+), (2) the ALG-targeted *5-HT*_*2A*_*R* RNAi knockdown (*w*^*1118*^;* Or42a- mCD8::GFP/+; GMR86E01-*Gal4/UAS-*5-HT*_*2A*_*R* RNAi), and (3) ALG-targeted *MMP-1* overexpression together with *5-HT*_*2A*_*R* RNAi knockdown (*w*^*1118*^;* Or42a- mCD8::GFP/UAS-MMP-1; GMR86E01-*Gal4/UAS-*5-HT*_*2A*_*R* RNAi). The VM7 innervation was visualized with Or42a::GFP labeling in 3D intensity images and the VM7 innervation volume quantified to measure the extent of pruning in all 6 genotypes and treatment conditions (Fig 8).

Controls show the expected pruning of the VM7 innervation with EB experience compared to the vehicle control (Fig 8A, top). As previous results above, *5-HT*_*2A*_*R* RNAi targeted only to ALG completely blocks this experience-dependent synaptic glomerulus pruning (Fig 8A, middle). The important new finding is that ALG-targeted *MMP-1* overexpression in combination with *5-HT*_*2A*_*R* RNAi (designated + *MMP-1* OE) effectively restores the pruning (Fig 8A, bottom). The extent of experience-dependent pruning is indistinguishable between the *MMP-1* OE condition and transgenic controls. Quantification of the VM7 innervation volume comparing oil vehicle and EB experience conditions shows a significant decrease in controls (2.6-fold; Fig 8B, left, green) and a similar significant decrease with *MMP-1* OE (3.3-fold; Fig 8B, blue). There remains no significant change in innervation volume between vehicle and EB experience conditions with ALG *5-HT*_*2A*_*R* RNAi (Fig 8B, red). There is a significant lost innervation volume (3.12-fold decrease) between EB conditions of *5-HT*_*2A*_*R* RNAi and *MMP-1* OE, but no significant difference between the *MMP-1* OE condition and the transgenic control, demonstrating a full restoration of experience-dependent synaptic glomerulus pruning. We conclude that ALG MMP-1 overexpression restores pruning prevented by knockdown of 5-HT_2A_R signaling. This mechanism suggests glia-to-glia serotonergic communication with 5-HT_2A_ receptor activation upstream of MMP-1 induction is essential for experience-dependent synaptic glomerulus pruning.

## Discussion

We discover a serotonergic signaling mechanism between two glial classes needed for experience-dependent synapse pruning: EG (serotonin production) and ALG (5-HT_2A_ reception). New *Drosophila* connectome FlyWire mapping [65] reveals complete synaptic partner connectivity for this brain circuit remodeling (Fig 1). Neuron-to-glia signals orchestrating such synaptic pruning are relatively well known [66,67], but glia-to-glia signaling is a largely unexplored frontier [1]. Mammalian glial studies have revealed intra-class signaling that mediates synapse pruning [68], but inter-class signaling is much less known. Synapse elimination is the end result of hierarchical activation signaling that ultimately results in synaptic destabilization and/or direct synapse phagocytosis by glia [69,70]. Intercellular “find me” signals guide glial infiltration [47,71,72], and subsequent “eat me” signals target precise phagocytosis [1,15]. Unlike neuron-to-glia signals, there is an underemphasis on glial-to-glia signaling and pruning coordination across different glia classes. Serotonergic signaling is well-known to regulate circuit formation and juvenile brain circuit remodeling in mammals [73,74]. Glial serotonin signaling is required for olfactory experience-dependent synaptic pruning in the juvenile *Drosophila* brain [21]. We here discover separable requirements for serotonin signaling and 5-HT_2A_ receptor function in two interacting glial classes, demonstrating a glia-to-glia class serotonergic signaling mechanism essential for highly precise synapse pruning in a temporally-restricted critical period and for re-opening this capacity at maturity.

We find glia infiltration into target synaptic neuropil is entirely by EG, revealing a novel glial class-restricted, experience-dependent mechanism (Fig 2A). During glial activation, glial membrane projections extend into the synaptic neuropil, but glial somata remain at the outer bounds of the AL, as shown with glia nucleus staining. Glia send their projections into input experience-targeted synaptic glomeruli for precise pruning via activation of input-specific signaling cascades [75]. For example, critical period experience induces odorant dose-dependent ERK activation in glia, with glial-targeted ERK loss impairing circuit-localized synaptic neuropill infiltration [76]. Glia also employ Draper (mammalian MEGF10) receptor activation, Basket (mammalian JNK) phosphorylation and nuclear translocation, and transcriptional upregulation of Cheerio (mammalian FLNA), to guide glial F-actin cytoskeleton dynamics for glial infiltration [15]. Consistent with this study, the Draper receptor requirement is solely in EG. The mechanism is conserved with injury responses, where a soma phagosome mediates degradation and recycling of the engulfed material, while projection infiltration acts as the mechanism of targeted engulfment and directed phagocytosis [77]. For synaptic pruning, this separable process means experience-dependent infiltration into EB-responsive VM7 synaptic glomeruli (Fig 2A), with synapses eliminated in a class-specific mechanism. Future studies will investigate integration of signaling cascades directing glial infiltration to targeted synapses for removal and degradation in separate cellular locations.

We discover glial 5-HT_2A_ receptors are required for experience-driven targeting, with glial *5-HT*_*2A*_*R* RNAi completely blocking projection infiltration into the EB-responsive synaptic glomeruli (Fig 2B and 2C). In mammalian cultures, neuronal 5-HT receptors trigger cytoskeleton remodeling for growth guidance during synaptogenesis and plasticity [78]. However, nothing is known about 5-HT receptors in glial projection guidance. Mammalian glia fractalkine (CX3CR1) signaling mediates infiltration in response to neuronal activation by injury or pathogens [79]. The signaling cascades regulated by cytokines are a potential link to glial serotonergic requirements [80]. Although these mechanisms are a response to neuronal injury or pathogenesis, the glial synaptic pruning process is thought to be conserved with these pathways [15]. In *Drosophila*, glial ephrin/Eph receptor signaling is also reportedly implicated in glial infiltration [81]. This independent mechanism for glial infiltration mirrors the requirement for 5-HT/5-HT_2A_R signaling in experience-dependent critical period synaptic pruning. We find glial *5-HT*_*2A*_*R* RNAi blocks glial infiltration entirely (Fig 2B and 2C), whereas EphA receptors provide spatial cues that appear to only modulate neuronal plasticity [81]. Nevertheless, together these findings suggest glial signals can orchestrate glial projection infiltration in mechanisms required for neuronal remodeling. Future studies are needed to further dissect glial class-specific requirements of 5-HT_2A_R signaling in the directional glial infiltration for experience-dependent synaptic pruning and provide insights into precise synapse elimination in sculpting juvenile brain circuitry.

We find that serotonin synthesis via tryptophan hydroxylase is also required only in EG, with cell-targeted RNAi blocking critical period experience-dependent synaptic glomeruli pruning (Fig 3). Glia phagocytosis is the primary means of neuronal pruning in both sensory experience-dependent and injury-induced mechanisms [1,66,67]. The different glial classes perform distinct pruning jobs [82,83]. In the juvenile *Drosophila* olfactory critical period, EG are the sole phagocytes responding to odorant experience [15]. The finding that this terminal endpoint glial phagocyte class is the source of serotonin signaling is surprising, with the circuit-localized serotonergic response driven solely by Or42a OSN activation [21]. In mammals, serotonergic signaling is also linked to glial functions, with activated glia triggering alternative tryptophan metabolism to reduce serotonin availability within the brain [84]. However, studies of serotonergic disease dysfunction often fail to consider possible glial signaling. For example, juvenile mice show reduced serotonin-mediated calcium signaling within astrocytes, which results in aberrant synaptic plasticity [26]. Nevertheless, glial functions and glia class interactions remain understudied. We find that EG *Trhn* RNAi completely blocks critical period synaptic glomerulus pruning (Fig 3). Future studies could test if experience-dependent serotonin signaling is solely from EG, with brain 5-HT levels quantified for EG-targeted *Trhn* RNAi. To further understand the requirement of glia-to-glia serotonergic signaling, we next tested glia class-specific 5-HT receptor roles.

We find astrocytic 5-HT_2A_ receptors alone are required for experience-dependent synaptic glomeruli pruning in the critical period (Fig 4). Importantly, EG are the sole synaptic phagocytes responding to early experience [15]. These paired class-specific requirements suggest a model in which ALG and EG are differentially required for the directional precise pruning of targeted synapses. This in turn suggests cooperative glial interactions and demonstrates a novel requirement for communication between different glial classes. Glia-to-glia signaling is a newer finding; however, precedents do exist. Mammalian glia produce pro-inflammatory cytokines, tumor necrosis factor alpha, interleukin-1β, and interferon-γ, to drive glial phagocytosis in response to injury or infection [85,86]. Moreover, mammalian astrocytes release CCL2/CXCL10 chemokines to signal glial attraction to sites of inflammation [87]. In Alzheimer’s disease models, a positive feedback signaling loop between microglia and astrocytes drives the inflammatory response, suggesting glia-to-glia communication for cooperative neuronal clearance [82]. Similarly, serotonin may signal from phagocytic glia (EG) to ALG to coordinate a cooperative phagocytosis mechanism. While the above examples are from injury or disease contexts, they provide a comparison for considering phagocytic glia producing a signal that works in a feedback mechanism in astrocytes during synaptic pruning in response to critical period experience. We propose serotonin signaling removes a protective glial block to guide pruning specificity.

We discover that astrocytic 5-HT_2A_ receptors are not only necessary for synaptic pruning, but are sufficient to rescue critical period experience-dependent synapse pruning in an otherwise global *5-HT*_*2A*_*R* null mutant (Fig 5). In neurons, the 5-HT_2A_ receptor is known to drive formation of serotonergic circuits via autoregulation [17,24], both during brain development and in adult learning/memory cascades [88,89]. The 5-HT_2A_ receptor distribution predicts plasticity capacities in adult mammalian brain [90], which suggests a connection between the receptor availability and synaptic signaling. In the *Drosophila* juvenile brain olfactory critical period, glial 5-HT_2A_ receptors have a rate-limiting role in the extent of experience-dependent synapse pruning [21]. The requirement of ALG activation for this synaptic pruning is entirely novel. Glial 5-HT_2A_ receptor upregulation occurs in the inflammatory response to traumatic brain injury [91], linked to serotonergic cognitive impairments [92], suggesting 5-HT_2A_Rs may drive localized synaptic changes. Our results here indicate that 5-HT_2A_Rs required only in astrocytes allows precise local synapse pruning in response to critical period experience. Thus, restoring this 5-HT_2A_ receptor signaling only in ALG is sufficient to rescue experience-dependent synaptic glomeruli pruning (Fig 5). Isolating this 5-HT_2A_R requirement to a single glial class may help answer the broad-spectrum question of how glial cells enact high-level specificity to a broad and diverse range of environmental sensory input, while enabling correct synaptic connectivity remodeling to optimize juvenile brain circuits.

We find astrocytic 5-HT_2A_ receptors are not only necessary and sufficient for critical period pruning, but that conditional 5-HT_2A_ receptor expression in adult astrocytes acts to re-open “critical period-like” experience-dependent synapse pruning at maturity (Fig 6). Synaptic plasticity in adults allows learning and memory formation; however, large-scale connectivity changes are largely prohibited at maturity [93]. Both long-term potentiation and depression can involve synapse formation and elimination [94,95], but operate on a smaller scale. Extensive remodeling is presumed to be blocked following critical period closure to insulate mature circuit properties [96,97], which are believed vital for higher-order cognition, but this poses an obstacle in treating trauma, injury, and disease. It is therefore a priority to find ways to re-open the heightened state of “critical period-like” remodeling to provide treatments for many circuit dysfunction conditions, such as autism spectrum disorder [98], schizophrenia, and Alzheimer’s disease [99,62]. While serotonergic psychoactive drugs (e.g., LSD and ketamine) have shown promise in elevating such synaptic remodeling capacities, specificity is a substantial concern [100]. Critical period closure is thought to protect against large-scale connectivity changes, so re-opening this ability without sufficient specificity protections is likely quite detrimental. Such specificity could possibly be derived from 5-HT_2A_R activation within specific glial classes to activate experience-dependent synapse pruning (Fig 6). Thus, we propose astrocyte-targeted serotonergic signaling as a potential new intervention strategy.

We discover that critical period sensory experience induces MMP-1 expression within ALG (Fig 7). Since serotonergic 5-HT_2A_R signaling activates MMP extracellular proteolytic enzymes [39], this provided a potential mechanism to explain the glia-to-glia serotonin signaling role in experience-dependent synaptic pruning. A primary restriction to large-scale connectivity remodeling is ECM synapse stabilization [101,102], with glial-mediated ECM changes implicated in both the projection infiltration and subsequent phagocytosis modulating this synapse stability [103,104]. In *Drosophila*, the glial *Draper* engulfment receptor (mammalian MEGF-10) initiates ECM remodeling to enable glial phagocytosis function [41]. In injury models, *Draper* receptor activation triggers the upregulation of glial MMP-1, however the mechanistic requirement for MMP-1 in other forms of glial infiltration remains unknown. Here, we show MMP-1 is induced in ALG for juvenile brain infiltration. We find MMP-1 is absolutely required in the ALG for experience-dependent synaptic pruning (Fig 7). In mammals, MMPs are long associated with responses to stroke-induced inflammation, as well as juvenile brain experience-dependent primary visual cortex remodeling plasticity [105]. Importantly, secreted MMP-9 overexpression in mice has been shown to re-open ‘critical period-like’ synaptic remodeling at maturity [106,107]. Here, we show secreted MMP-1 is an essential component of *Drosophila* experience-dependent synaptic pruning, indicating a well-conserved mechanism enabling brain circuit plasticity.

We discover a direct connection between glial 5-HT_2A_R and MMP-1 roles, with MMP-1 overexpression compensating for 5-HT_2A_R loss to restore experience-dependent synaptic pruning (Fig 8). 5-HT_2A_R signaling is well known to activate ECM-digesting MMP extracellular endopeptidases via ERK1/2 signaling [39], and we have also recently discovered that experience-dependent ERK signaling is required for synapse pruning [39,76]. In injury models, 5-HT_2A_R activation upregulates MMPs for ECM remodeling [37]. We postulate that glial 5-HT_2A_R signaling in critical period experience-dependent synaptic glomerulus pruning similarly upregulates glial secreted MMP-1 to enable circuit-localized ECM remodeling for targeted glial infiltration. Our data support the hypothesis that MMP-dependent ECM remodeling allows large-scale synaptic connectivity alterations [35,108]. We add the novel dimension that temporally-restricted glial 5-HT_2A_R signaling delineates the critical period window of MMP function in experience-dependent synapse elimination. We find glial infiltration is blocked by loss of glial 5-HT_2A_R signaling (Fig 2), and further testing MMP-1 requirements in this mechanism is an important future goal. Future studies will examine experience-dependent glial infiltration with targeted MMP-1 knockdown, and MMP-1 roles during 5-HT_2A_R signaling deficient loss of glial infiltration. In conclusion, the work reported here reveals novel glia-to-glia class serotonergic 5-HT_2A_R signaling that induces MMP-1 upregulation as an important and overlooked control mechanism driving experience-dependent, circuit-localized synapse pruning in brain circuits.

## Methods

### *Drosophila* genetics

Animals were maintained at 25°C in a 12:12-hour light/dark incubator on standard food. The Gal4 driver lines were: pan-glial *repo-*Gal4 (RRID: BDSC 7415) [109], ALG *GMR86E01*-Gal4 (RRID: BDSC: 45914) [110], EG *GMR56F03*-Gal4 (RRID: 39157) [111], and CG *GMR54H02*-Gal4 (RRID: 45784) [112]. The UAS responder lines were: membrane markers UAS-*mCD8*::*GFP* (RRID: BDSC 5137) [113] UAS-*mCD8*::*RFP* (RRID: BDSC 32219) [114], and UAS-Luciferase (RRID: BDSC 35789) [115]; the serotonergic UAS*-Trhn* RNAi (RRID: BDSC 25842) [111], UAS-*5HT*_*2A*_*R* RNAi (RRID: BDSC 31882) [116], and UAS-*5-HT*_*2A*_*R* OE (RRID: BDSC 24504) [117]; the MMP pathway *UAS-MMP1 RNAi* [32,118], *UAS-MMP1* OE (RRID: BDSC:58700) [32,118,119], UAS-*MMP-2* RNAi (RRID: BDSC:31371) [32,118], and also *UAS-Dicer-2* (RRID: BDSC 24651) [119]. The temperature-sensitive (ts) Gal80 repressor was α-*tubulin-Gal80*^ts^ (RRID: BDSC 86328) [64]. Direct promoter fusion line was *Or42a-mCD8*::*GFP* [44,120]. The *5-HT*_*2A*_ *receptor* null mutant line was *w*^*1118*^; *5-HT*_*2A*_*R*^*null*^ (RRID: BDSC 84444) [121]. The genetic background control was *w*^*1118*^ (RRID: BDSC 3605), and multiple transgenic controls used were: (1) *w*^*1118*^;* UAS-mCD8*::*RFP/+; repo-*Gal4*/mCD8-Or42a::GFP,* (2) *w*^*1118*^;* Or42a-mCD8*:*GFP*/+; *repo-*Gal4*/*+, (3) *w*^*1118*^;* Or42a-mCD8*:*GFP/+; UAS-Trhn* RNAi*/+*, (4) *w*^*1118*^;* Or42a-mCD8*:*GFP/+; UAS-5-HT*_*2A*_*R* RNAi*/+*, (5) *w*^*1118*^;* Or42a-mCD8*::*GFP/+; GMR86E01-*Gal4/+, *(6) w*^*1118*^;* UAS-5-HT*_*2A*_*/*α-*tubulin-Gal80*^ts^; *Or42a-mCD8*::*GFP*, (7) *w*^*1118*^;* Or42a-mCD8::GFP/+; GMR86E01-*Gal4 (ALG)/+, (8) (*w*^*1118*^;* Or42a-mCD8::GFP/+; GMR86E01-*Gal4 (ALG)/+, and (9) *w*^*1118*^;* Or42a-mCD8*::*GFP/UAS-Dicer-2; UAS-MMP-2 RNAi/+.* Animals of both sexes were used in all studies.

### Odorant exposure

Odor experience treatments were done as previously described [122]. Briefly; animals were developmentally staged, and then sorted as late-stage pupae into separate vials based on sex and genotype. A fine wire stainless steel mesh was secured with taped Parafilm over the top of the vials. The vials were placed in an airtight 3,700 ml Glasslock container with 1 ml mineral oil vehicle (100%; Sigma-Aldrich) or 25% EB odorant (Sigma-Aldrich; % v/v EB in mineral oil) in a 1.5-ml microcentrifuge tube centered in the odorant exposure chamber. The odorant chambers were placed into temperature-controlled incubators (25°C) on a 12-hour light/dark cycle for all the non-temperature-sensitive animals. Newly eclosed flies were rapidly transferred to new vials in exposure chambers with freshly made odorants (as above), 4–6 hours after placing the vials into the chambers. The animals were then maintained in the odorant exposure chambers in incubators for a further 18–20 hours (24 hours total), and then immediately processed for brain immunocytochemistry [122].

### Conditional transgenics

For all temperature-sensitive Gal80 (Gal80^ts^) trials, developmentally staged animals were sorted as dark pupae into separate vials based on age, sex, and genotype. All animals used were raised at 18°C restrictive temperature with 12-hour light/dark cycle to either a juvenile critical period age (2–4 days, S5 Fig) or to mature adult age (14–16 days, Fig 6) and then transferred to experimental temperature (18°C restrictive or 28°C permissive temperature) for odorant exposure. As above, animals were exposed in vials with a fine wire stainless steel mesh top in airtight Glass- lock containers with either the 1 ml vehicle control only (100% mineral oil; Sigma-Aldrich) or the 25% EB odorant (Sigma-Aldrich; % v/v EB in mineral oil). The animals were maintained in the odorant exposure chambers in temperature-controlled incubators for 24 hours before immediately being processed for brain immunocytochemistry [122].

### Immunocytochemistry imaging

Post-exposure animals were anesthetized in 70% ethanol for 1–2 min. Brains were dissected using sharpened forceps (Dumont #5, C shape) in 1× phosphate-buffered saline (PBS; Invitrogen). Brains were fixed for 30 min at room temperature (RT) in 4% paraformaldehyde (PFA; EMS 15714) in 4% sucrose PBS. Fixed brains were washed 3× with PBS and then blocked for 1.5–2 hours at RT or overnight (12–16 hours in 4°C) with 1% BSA (Sigma-Aldrich) in 0.2% Triton X-100 in PBS (PBS-T; Fisher Chemical). The brains were incubated with all primary antibodies diluted in 0.2% BSA in PBS-T at 4°C overnight. The primary antibodies used were: chicken anti-GFP (Abcam, 13970; 1:1,000), rat anti-RFP (Chromotek, 5F8; 1:1,000), and rat anti-DNEX-8 (Developmental Studies Hybridoma Bank (DSHB); 1:50), mouse anti-MMP1 (DSHB, 5H7B11; 1:50), mouse anti-MMP1 (DSHB, 3A6B4; 1:50), and mouse anti-MMP1 (DSHB, 3B8D12; 1:50). All three anti-MMP1 antibodies were used together in a 1/1/1 mixture, as previously described [123]. The brains were washed 3× for 20 min each with PBS-T and then incubated overnight with fluorescently conjugated secondary antibodies. The secondary antibodies used were: AlexFluor-488 goat anti-chicken, AlexFluor-488 goat anti-mouse, AlexaFluor-546 goat anti-rat, and AlexaFluor-546 donkey anti-rat (all used at 1:250). The brains were washed in PBS-T 3× for 20 min each, followed by PBS 1× for 20 min. Brains were mounted onto glass slides (75 × 25 mm, 0.9–1.06 mm; Corning) with a glass coverslip (No. 1.5H, Carl Zeiss) in Fluoromount-G (EMS 17984–25). Double-sided adhesive tape (Scotch) was used to raise coverslips over the brains, and clear nail polish (Sally Hansen) was used to seal coverslips. Images were collected on a 510 META laser-scanning confocal microscope (Carl Zeiss) with a 63× oil-immersion objective. Images were collected at 1,024 × 1,024 resolution with a Z-slice thickness of 0.75 μm. The microscope and imaging settings were kept constant within every experiment (exact settings can be found in Protocol Exchange).

### Quantification measurements

All measurements were done blind to both genotype and experience conditions using the ImageJ Blind Analysis Tool plug-in. To quantify EG and pan-glial *repo*-Gal4 driving UAS-*mCD8*::*RFP* membrane innervation values, the weighted sum of all pixels was used, which adds together all the pixels in each slice at each position. Brightness values <50 were dropped to account for imaging background. These values were then normalized to the mineral oil control in each analysis. For glial-specific infiltration, a defined ROI was made from the borders of the VM7 glomerulus and innervation volume was quantified by lasso perimeter measurements from the sum slices Z-projection using the following equation: [*volume* (*μm*^3^) = *area* (*μm*^2^) *× slice thickness × total number of slices*]. For Or42a innervation volumes, an ROI was defined for the VM7 glomerulus quantified by lasso perimeter measurements from the sum slices Z-projection using the following equation: [*volume* (*μm*^3^) = *area* (*μm*^2^) *× slice thickness × total number of slices*]. To quantify MMP-1 intensity values, the weighted sum of all pixels was used, which adds together all the pixels in each slice at each position. Brightness values <50 were dropped to account for imaging background. Data from all the combined biological replicates were maintained as a raw measurement point spread, with blinded quantification to ensure normality across all trials [122].

### Statistical analyses

All statistical analyses were performed with Prism software (GraphPad version 9). All analyses were done using *n* = number of synaptic glomeruli, unless otherwise stated. All groups that met the criteria for parametric statistics were analyzed with unpaired two-tailed *t* tests. For data comparing >2 genotypes, a two-way ANOVA was used with odorant exposure and genotype as independent variables, followed by the Sidak’s multiple-comparisons test to compare the oil odorant vehicle and EB-exposure conditions within each genotype. Comparisons between 2 or more genotypes were analyzed by two-way ANOVA tests with a 5% alpha significance level. Data are presented in the figures as all individual data points and mean ± SEM. Significance in figures is indicated as *p* < 0.05 (*), *p* < 0.01 (**), *p* < 0.001 (***), *p* < 0.0001 (****), and *p* > 0.05 marked not significant (ns). The exact significance *p*-values for each comparison are given in the figure legends.

## Supporting information

S1 Fig Different transgenic Gal4 drivers for the three antennal lobe glial classes. Juvenile brain antennal lobe (AL) expression of the UAS-mCD8::GFP membrane marker (green) driven in three different glial classes: **A**, ensheathing glia (EG, R56F03-Gal4); **B**, astrocyte-like glia (ALG, R86E01-Gal4); and **C**, cortex glia (CG, R54H02-Gal4). Brains were dissected from animals 0 to 1 days post-eclosion (dpe). Scale bar: 10 μm. The data underlying this Figure can be found in S1 Data.
(TIFF)

S2 Fig Astrocyte and cortex glia show no infiltration with odorant experience. **A**, Astrocyte-like glia (top) GMR86E01-Gal4 and Cortex Glia (bottom) GMR54H02-Gal4 driven UAS-MCD8::RFP in antennal lobe 3D projection (depth color-coded scale, bottom right), with 24-hour (0–1 dpe) exposure to vehicle control (oil, left) or odorant experience (EB, right). VM7 synaptic glomeruli shown in white. **B**, Quantification of glial in VM7 shows no significant infiltration for astrocyte-like glia (blue, *p* = 0.525) or cortex glia (red, *p* = 0.829) with EB experience. All data points with mean ± SEM. Not significant; *p* > 0.05 (ns). The data underlying this Figure can be found in S1 Data.
(TIFF)

S3 Fig Glial repo-Gal4 driver effectiveness not diluted with dual UAS constructs. Experience-dependent glial VM7 infiltration is indistinguishable with single UAS control (*w*^*1118*^;* UAS-mCD8::RFP/+; repo-Gal4/mCD8-Or42a::GFP*) and double UAS control (*w*^*1,118*^; UAS-mCD8::RFP/mCD8-Or42a::GFP; *repo*-Gal4/UAS-luciferase). Oil controls are not significantly different (*p* = 0.999) and EB treatments are not significantly different (*p* = 0.588). All data points with mean ± SEM. Not significant; *p* > 0.05 (ns). The data underlying this Figure can be found in S1 Data.
(TIFF)

S4 Fig Different glial transgenic Gal4 drivers express with comparable strength. Glial florescence intensity measured in juvenile brains with a single UAS construct driven by glial class-specific Gal4 drivers (*w*^*1118*^; UAS-mCD8::GFP/+; GMR56F03-Gal4 (EG)/+ or *w*^*1118*^; UAS-mCD8::GFP/+; *GMR86E01*-Gal4(ALG)/+) or two UAS constructs driven by glial class-specific Gal4 drivers (*w*^*1118*^; UAS-mCD8::GFP/UAS-mCD8::RFP; GMR56F03-Gal4 (EG)/+ or *w*^*1118*^; UAS-mCD8::GFP/UAS-mCD8::RFP; *GMR86E01*-Gal4(ALG)/+). Quantification shows no change with ensheathing glia Gal4 (*p* = 0.948) or astrocyte-like glia Gal4 (*p* = 0.502) drivers. All data points with mean ± SEM. Not significant; *p* > 0.05 (ns). The data underlying this Figure can be found in S1 Data.
(TIFF)

S5 Fig Conditional adult serotonin loss blocks experience-dependent pruning. **A**, Critical period experience-dependent synaptic glomerulus pruning with Gal80^ts^ active (top; 18°C, blue, permissive temperature) in *w*^*1118*^; tubulin-Gal80^ts^/Or42a-mCD8::GFP; UAS-*Trhn* RNAi/ GMR56F03-Gal4. Pruning is blocked by EG-specific conditional adult *Trhn* RNAi expression with Gal80^ts^ repressed (bottom; 28°C, red, restrictive temperature). 24-hour (0–1 dpe) vehicle control (oil, left) or odorant experience (EB, right). **B**, Or42a OSN innervation volume quantified at permissive 18°C (blue) and restrictive 28°C (red). Two-way ANOVA with Tukey’s multiple comparison tests show significant EB-induced pruning at permissive temperature (*p* = 8.830 × 10^−13^), but not when *Trhn* RNAi is driven in adult EG at restrictive temperature to cause no significant pruning (*p* = 0.9943). All data points with mean ± SEM. Significance: p < 0.0001 (****) and p > 0.05 (not significant; ns). The data underlying this Figure can be found in S1 Data.
(TIFF)

S6 Fig EG-specific MMP-1 RNAi does not block glial infiltration or pruning. **A**, Ensheathing glia in VM7 glomeruli (EG-Gal4 driven UAS-mCD8::RFP; heat-map) with ensheathing glia MMP-1 knockdown (*w*^*1118*^; GMR56F03-Gal4/UAS-MMP-1 RNAi; UAS-mCD8::RFP/UAS-Dicer-2) after 24-hour (0–1 dpe) exposure to vehicle control (oil, left) or odorant experience (EB, right) from 0 to 1 days post-eclosion (dpe). **B**, Quantification of glial infiltration into VM7 shows a significant increase with critical period EB experience (*p* = 1.672 × 10^−12^). **C**, Quantification VM7 innervation volume shows a significant decrease with critical period EB experience (*p* = 1.934 × 10^−10^). All data points with mean ± SEM. Significance indicated as *p* < 0.0001 (****). The data underlying this Figure can be found in S1 Data.
(TIFF)

S7 Fig MMP-2 plays no role in critical period experience-dependent pruning. **A**, Or42a neuron innervation of VM7 synaptic glomeruli shown with Or42a::GFP labeling (colored heat-map,16 LU scale; bottom right), co-labeled with anti-N-Cadherin (CadN) for visualization of all synaptic glomeruli (gray scale). Top row shows transgenic control of UAS-MMP-2 RNAi only (*w*^*1118*^; Or42a-mCD8::GFP/UAS-Dicer-2; UAS-MMP-2 RNAi/+). Bottom row shows glial *repo*-Gal4 driven MMP-2 RNAi (*w*^*1118*^; Or42a-mCD8::GFP/UAS-Dicer-2; UAS-MMP-2 RNAi/ *repo*-Gal4). Vehicle control (oil, left) or odorant experience (EB, right) for 24 hours from 0 to 1 dpe. **B**, Quantification of Or42a OSN innervation volume in *repo*-Gal4 control (green, left), driving MMP-1 RNAi (red, middle), or MMP-2 RNAi (blue, right). Two-way ANOVA with Tukey’s multiple comparison shows VM7 innervation pruning in *repo*-Gal4 control (*p* = 6.535 × 10^−11^) and driving MMP-2 RNAi (*p* = 6.551 × 10^−11^), but no significant pruning with MMP-1 RNAi (*p* = 0.523). All data points with mean ± SEM. Significance: *p* < 0.0001 (****) and *p* > 0.05 (not significant, ns). The data underlying this Figure can be found in S1 Data.
(TIFF)

S1 Data Data that underlies this paper. (XLSX)

## Abbreviations

AL: antennal lobe
ALG: astrocyte-like glia
CG: cortex glia
EB: ethyl butyrate
EG: ensheathing glia
ECM: extracellular matrix
LH: lateral horn
MB: mushroom body
MMP: matrix metalloprotease
OSN: olfactory sensory neuron
PBS: phosphate-buffered saline
PNs: projection neurons
RT: room temperature
VM7: ventral medial 7
