# Supplementary figures and images for: Glia-to-glia serotonin signaling directs MMP-dependent infiltration for experience-dependent synapse pruning
Source: PLoS Biol. 2025 Dec 1;23(12):e3003524. doi: 10.1371/journal.pbio.3003524 (PMC12668486; doi:10.1371/journal.pbio.3003524)

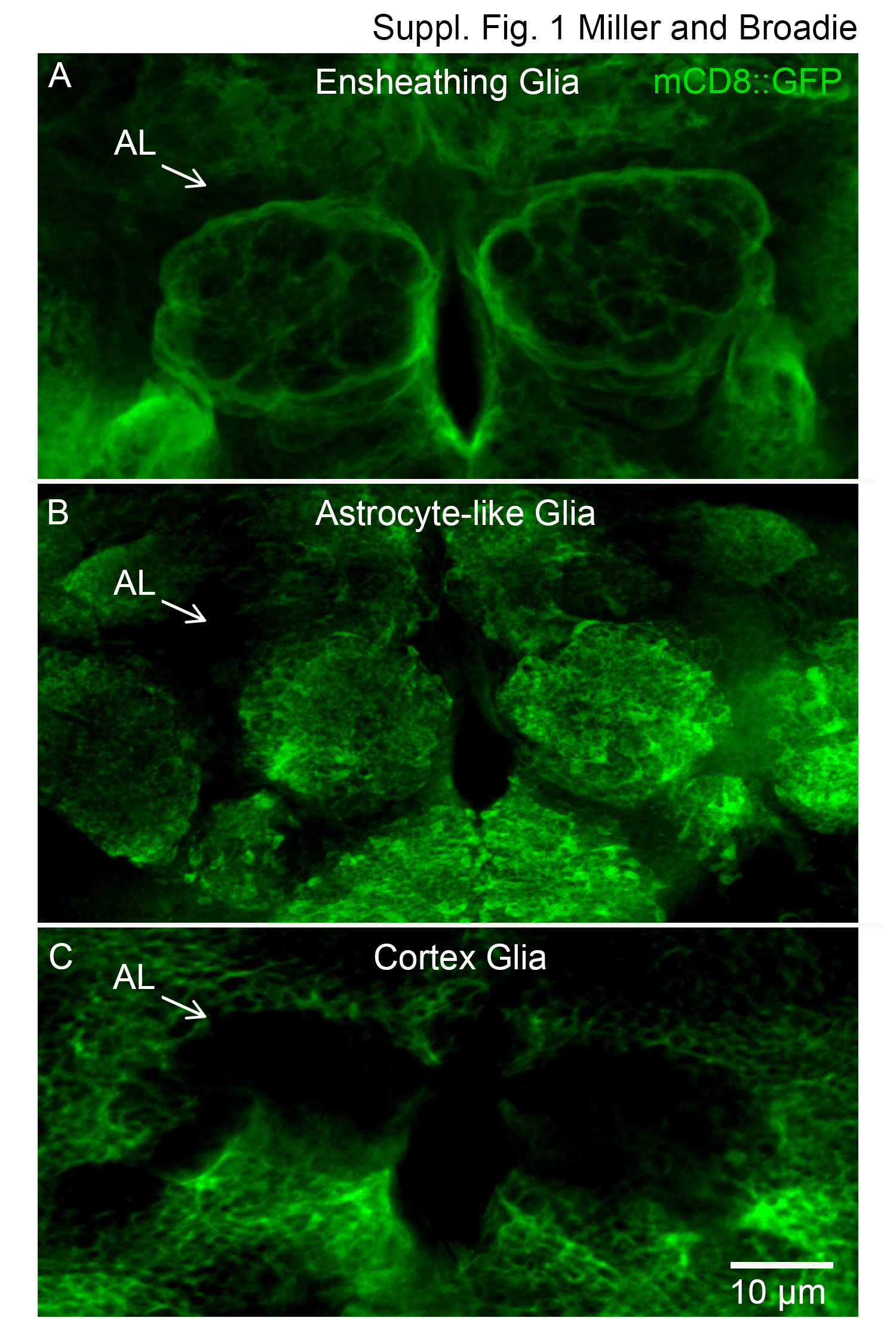

Supplement: S1 Fig — Juvenile brain antennal lobe (AL) expression of the UAS-mCD8::GFP membrane marker (green) driven in three different glial classes: A, ensheathing glia (EG, R56F03-Gal4); B, astrocyte-like glia (ALG, R86E01-Gal4); and C, cortex glia (CG, R54H02-Gal4). Brains were dissected from animals 0 to 1 days post-eclosion (dpe). Scale bar: 10 μm. The data underlying this Figure can be found in S1 Data. (TIFF) [file pbio.3003524.s001.tiff]

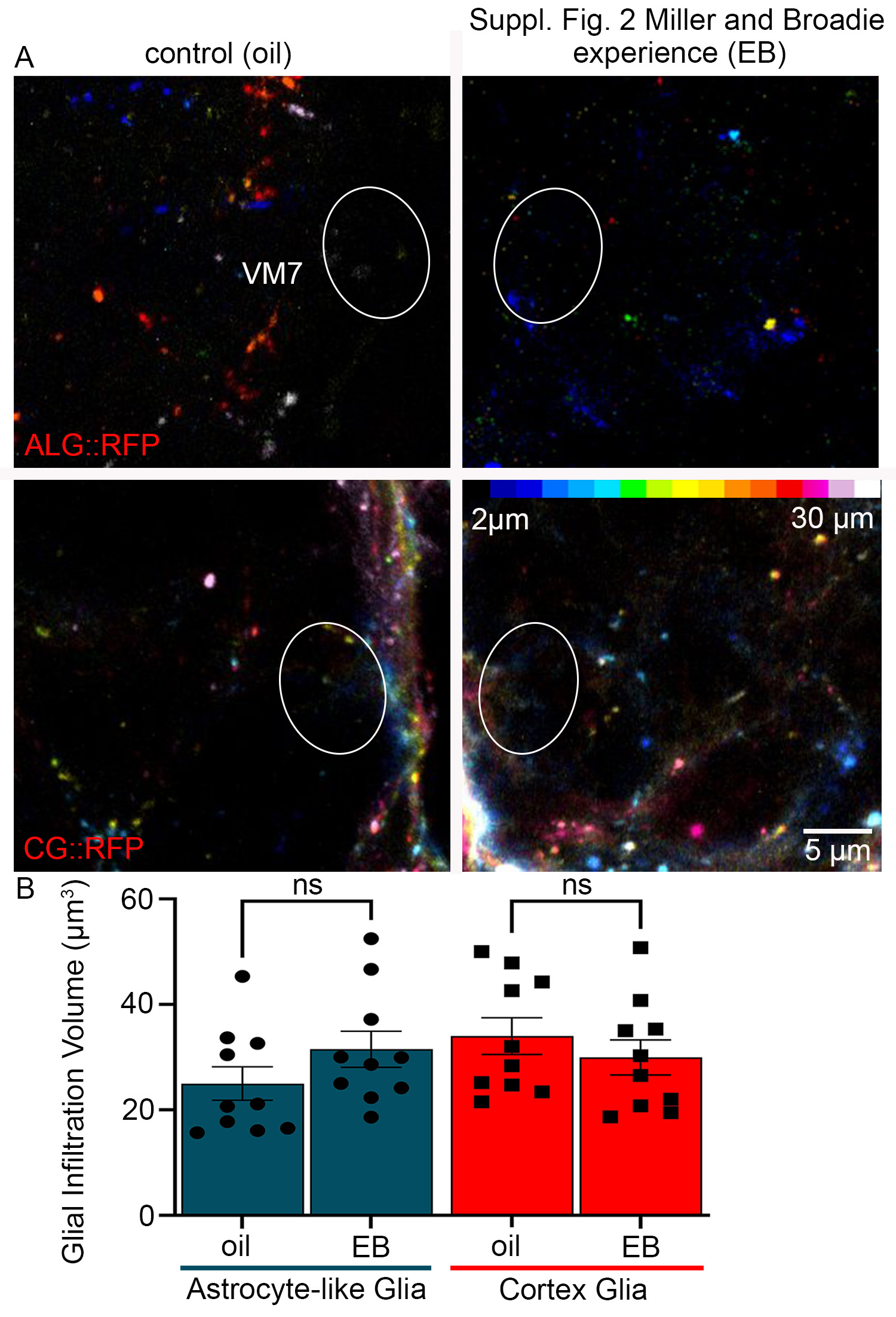

Supplement: S2 Fig — A, Astrocyte-like glia (top) GMR86E01-Gal4 and Cortex Glia (bottom) GMR54H02-Gal4 driven UAS-MCD8::RFP in antennal lobe 3D projection (depth color-coded scale, bottom right), with 24-hour (0–1 dpe) exposure to vehicle control (oil, left) or odorant experience (EB, right). VM7 synaptic glomeruli shown in white. B, Quantification of glial in VM7 shows no significant infiltration for astrocyte-like glia (blue, p = 0.525) or cortex glia (red, p = 0.829) with EB experience. All data points with mean ± SEM. Not significant; p > 0.05 (ns). The data underlying this Figure can be found in S1 Data. (TIFF) [file pbio.3003524.s002.tiff]

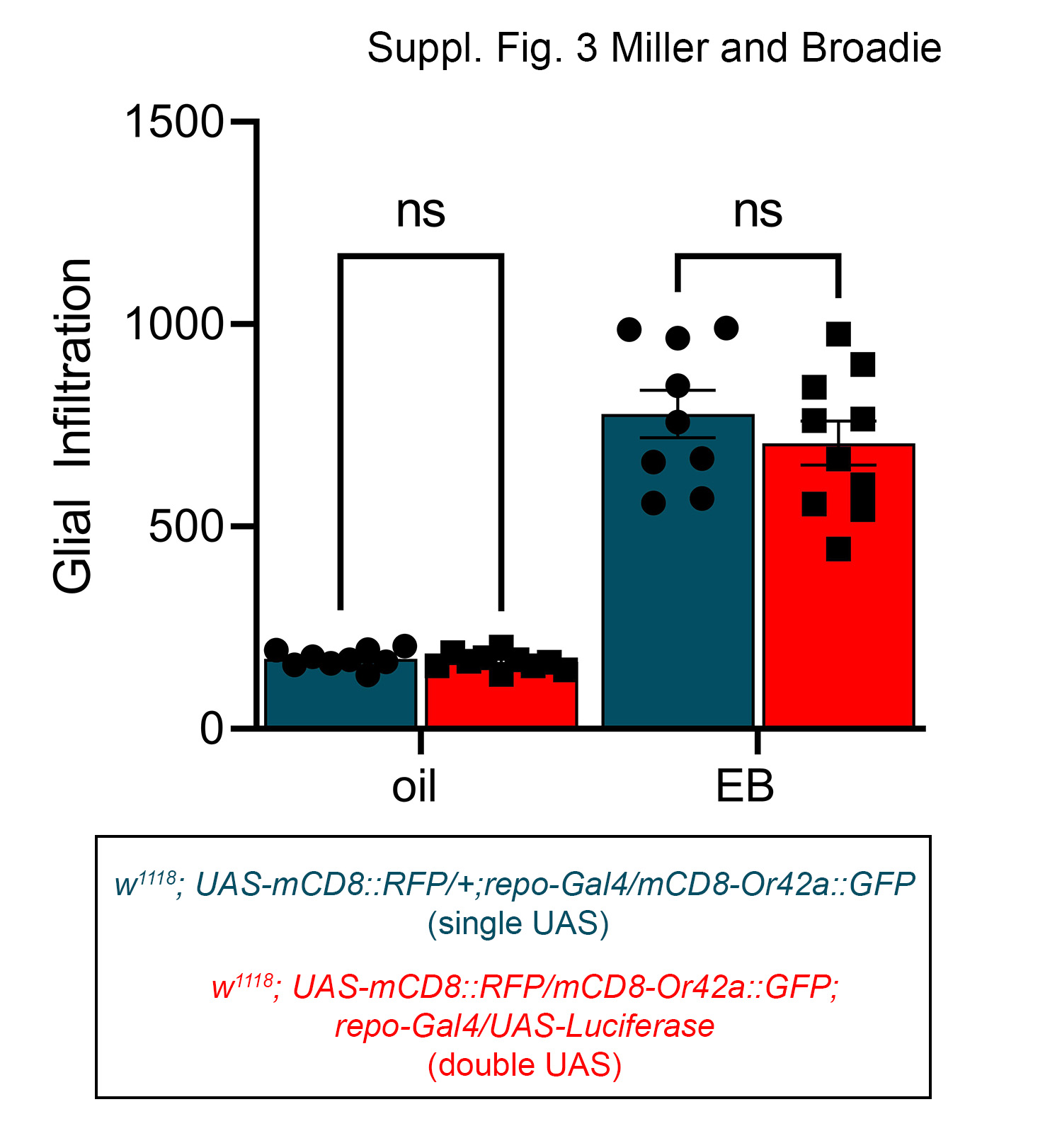

Supplement: S3 Fig — Experience-dependent glial VM7 infiltration is indistinguishable with single UAS control (w1118; UAS-mCD8::RFP/+; repo-Gal4/mCD8-Or42a::GFP) and double UAS control (w1,118; UAS-mCD8::RFP/mCD8-Or42a::GFP; repo-Gal4/UAS-luciferase). Oil controls are not significantly different (p = 0.999) and EB treatments are not significantly different (p = 0.588). All data points with mean ± SEM. Not significant; p > 0.05 (ns). The data underlying this Figure can be found in S1 Data. (TIFF) [file pbio.3003524.s003.tiff]

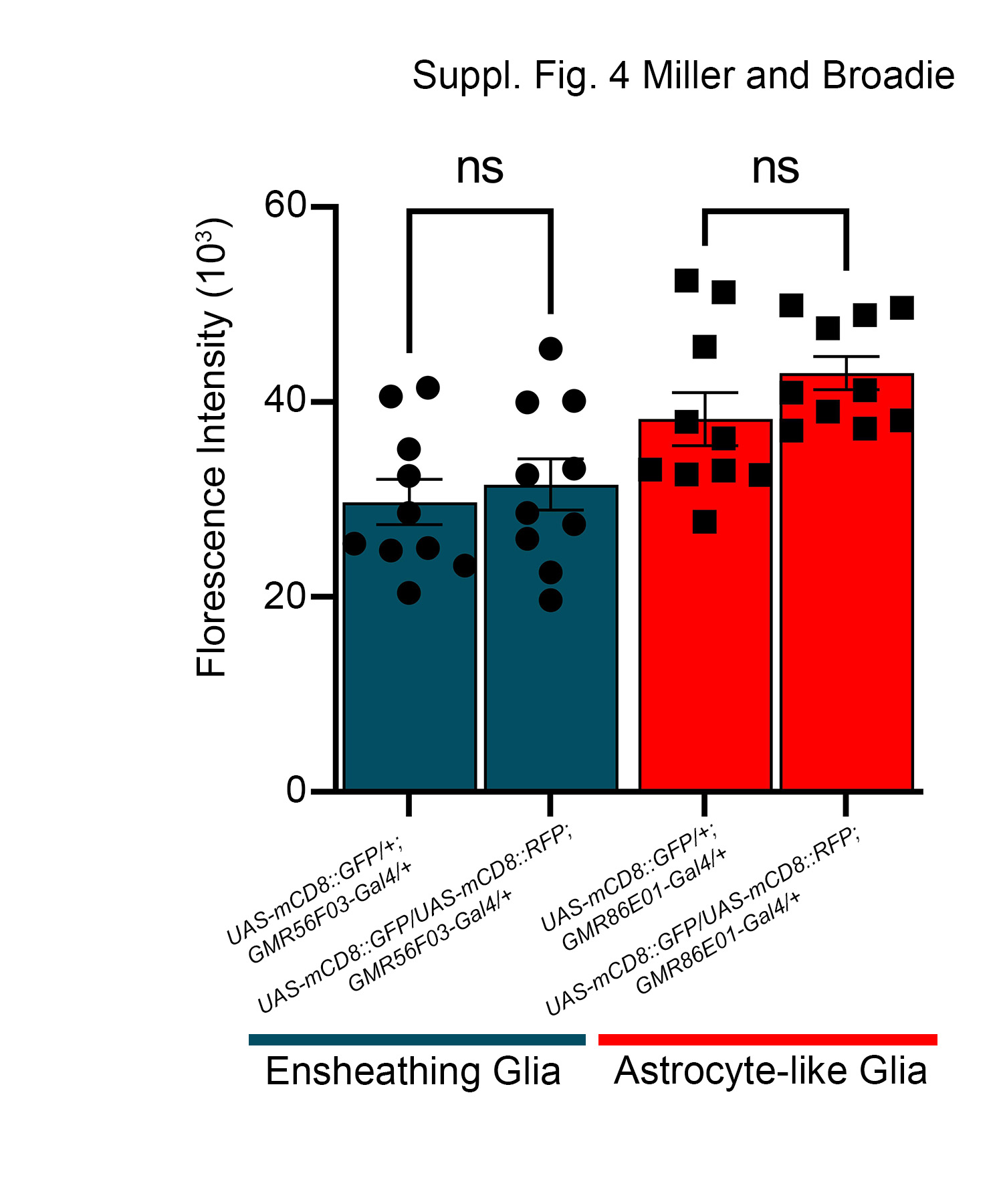

Supplement: S4 Fig — Glial florescence intensity measured in juvenile brains with a single UAS construct driven by glial class-specific Gal4 drivers (w1118; UAS-mCD8::GFP/+; GMR56F03-Gal4 (EG)/+ or w1118; UAS-mCD8::GFP/+; GMR86E01-Gal4(ALG)/+) or two UAS constructs driven by glial class-specific Gal4 drivers (w1118; UAS-mCD8::GFP/UAS-mCD8::RFP; GMR56F03-Gal4 (EG)/+ or w1118; UAS-mCD8::GFP/UAS-mCD8::RFP; GMR86E01-Gal4(ALG)/+). Quantification shows no change with ensheathing glia Gal4 (p = 0.948) or astrocyte-like glia Gal4 (p = 0.502) drivers. All data points with mean ± SEM. Not significant; p > 0.05 (ns). The data underlying this Figure can be found in S1 Data. (TIFF) [file pbio.3003524.s004.tiff]

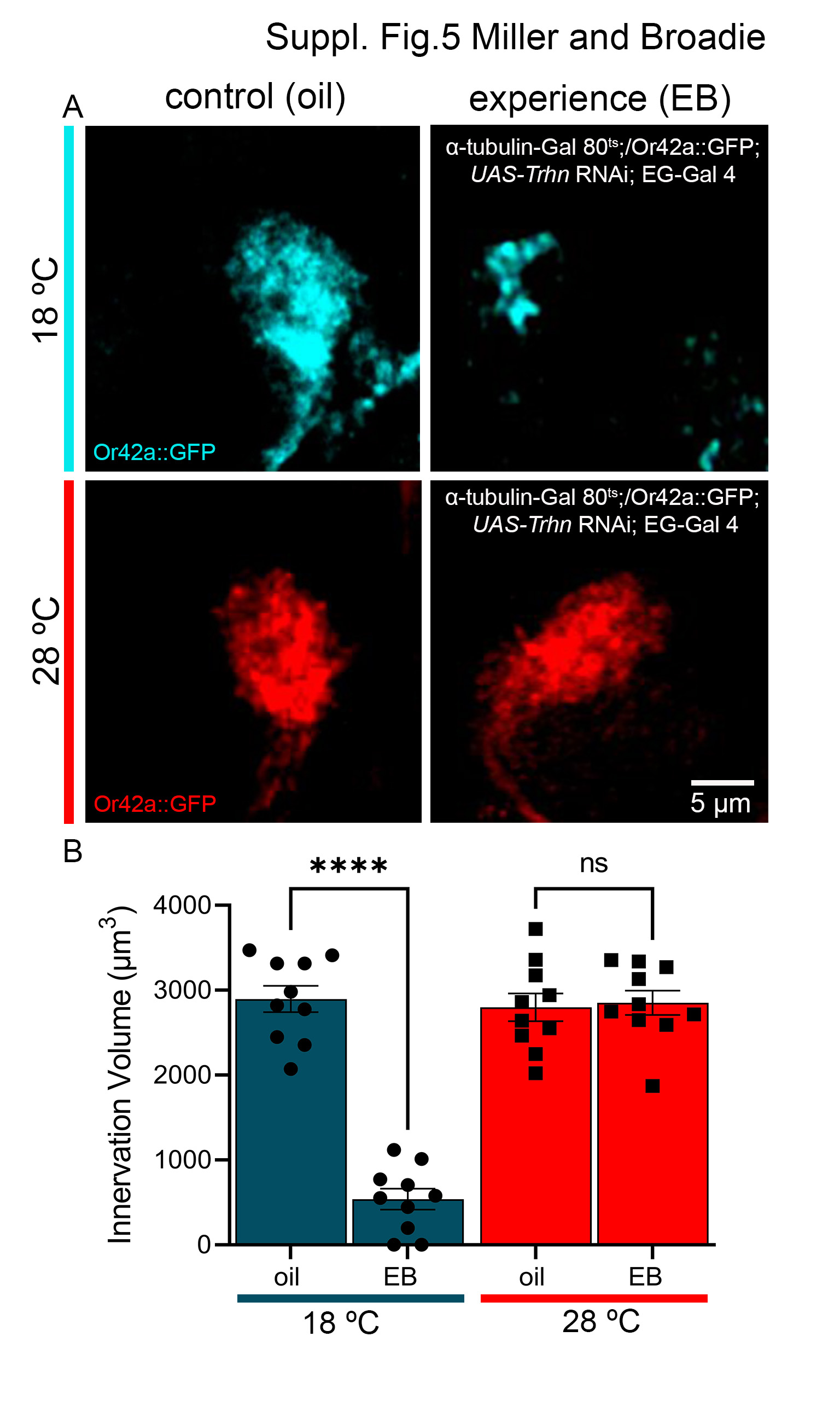

Supplement: S5 Fig — A, Critical period experience-dependent synaptic glomerulus pruning with Gal80ts active (top; 18°C, blue, permissive temperature) in w1118; tubulin-Gal80ts/Or42a-mCD8::GFP; UAS-Trhn RNAi/ GMR56F03-Gal4. Pruning is blocked by EG-specific conditional adult Trhn RNAi expression with Gal80ts repressed (bottom; 28°C, red, restrictive temperature). 24-hour (0–1 dpe) vehicle control (oil, left) or odorant experience (EB, right). B, Or42a OSN innervation volume quantified at permissive 18°C (blue) and restrictive 28°C (red). Two-way ANOVA with Tukey’s multiple comparison tests show significant EB-induced pruning at permissive temperature (p = 8.830 × 10^−13^), but not when Trhn RNAi is driven in adult EG at restrictive temperature to cause no significant pruning (p = 0.9943). All data points with mean ± SEM. Significance: p < 0.0001 (****) and p > 0.05 (not significant; ns). The data underlying this Figure can be found in S1 Data. (TIFF) [file pbio.3003524.s005.tiff]

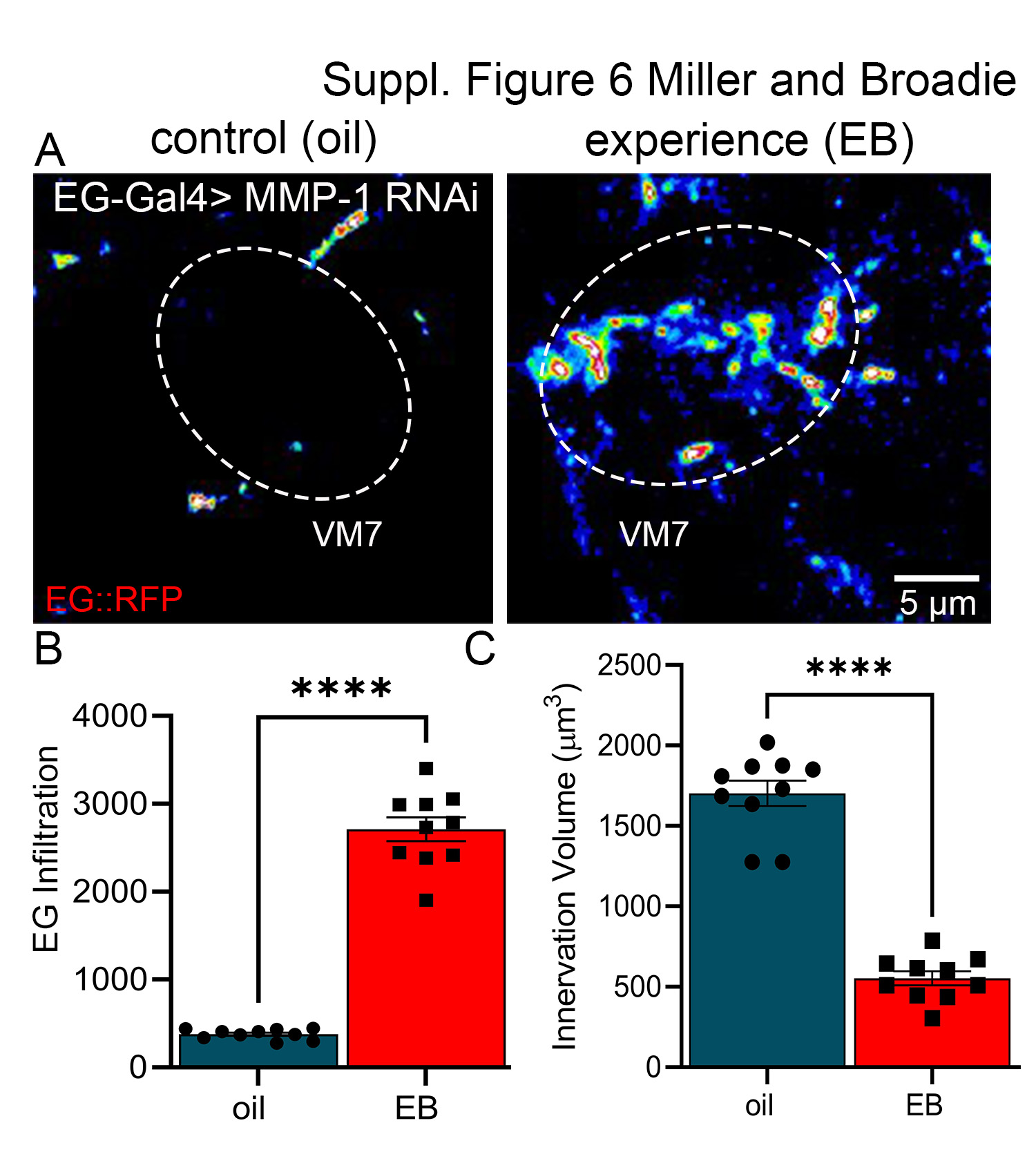

Supplement: S6 Fig — A, Ensheathing glia in VM7 glomeruli (EG-Gal4 driven UAS-mCD8::RFP; heat-map) with ensheathing glia MMP-1 knockdown (w1118; GMR56F03-Gal4/UAS-MMP-1 RNAi; UAS-mCD8::RFP/UAS-Dicer-2) after 24-hour (0–1 dpe) exposure to vehicle control (oil, left) or odorant experience (EB, right) from 0 to 1 days post-eclosion (dpe). B, Quantification of glial infiltration into VM7 shows a significant increase with critical period EB experience (p = 1.672 × 10^−12^). C, Quantification VM7 innervation volume shows a significant decrease with critical period EB experience (p = 1.934 × 10^−10^). All data points with mean ± SEM. Significance indicated as p < 0.0001 (****). The data underlying this Figure can be found in S1 Data. (TIFF) [file pbio.3003524.s006.tiff]

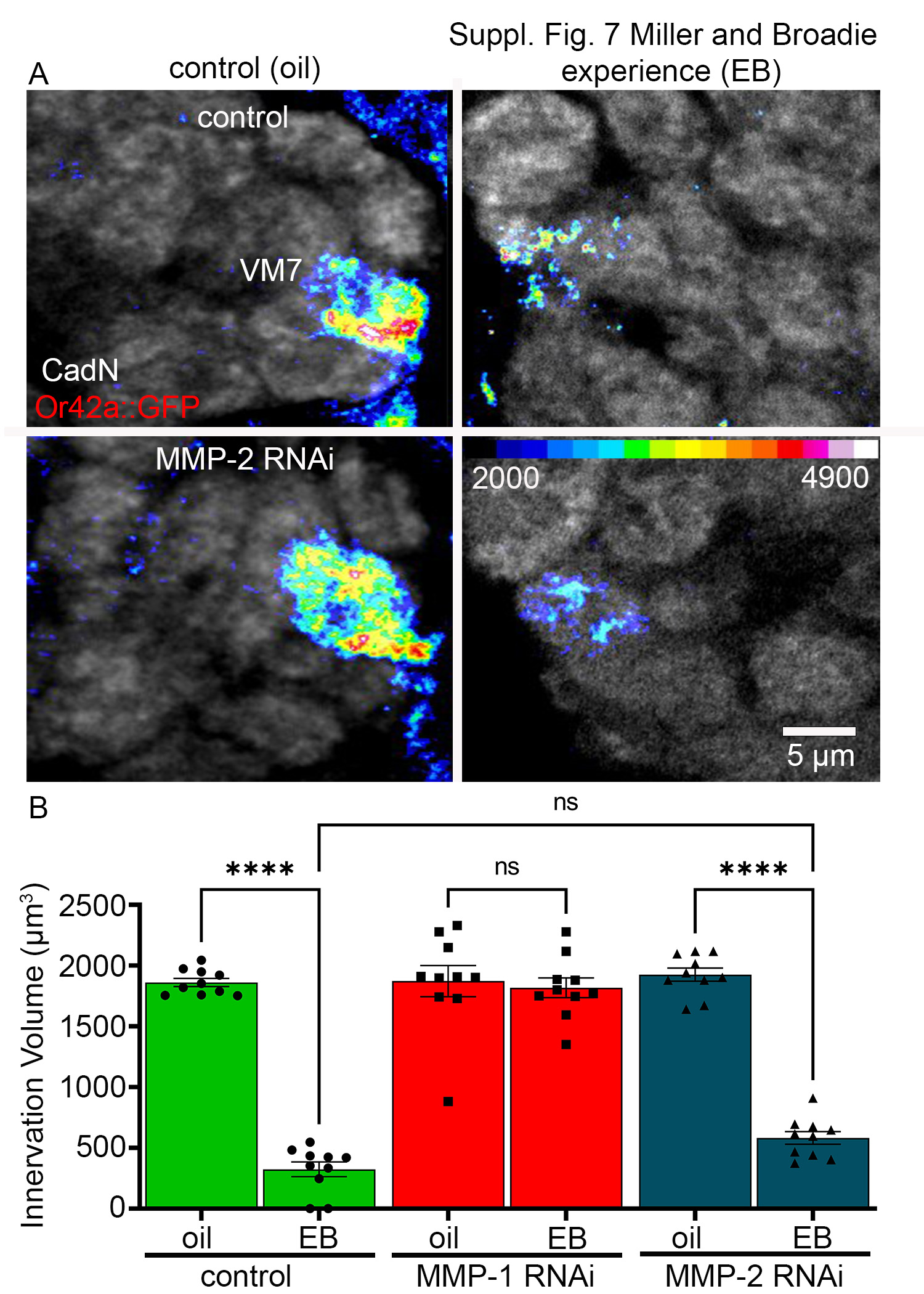

Supplement: S7 Fig — A, Or42a neuron innervation of VM7 synaptic glomeruli shown with Or42a::GFP labeling (colored heat-map,16 LU scale; bottom right), co-labeled with anti-N-Cadherin (CadN) for visualization of all synaptic glomeruli (gray scale). Top row shows transgenic control of UAS-MMP-2 RNAi only (w1118; Or42a-mCD8::GFP/UAS-Dicer-2; UAS-MMP-2 RNAi/+). Bottom row shows glial repo-Gal4 driven MMP-2 RNAi (w1118; Or42a-mCD8::GFP/UAS-Dicer-2; UAS-MMP-2 RNAi/ repo-Gal4). Vehicle control (oil, left) or odorant experience (EB, right) for 24 hours from 0 to 1 dpe. B, Quantification of Or42a OSN innervation volume in repo-Gal4 control (green, left), driving MMP-1 RNAi (red, middle), or MMP-2 RNAi (blue, right). Two-way ANOVA with Tukey’s multiple comparison shows VM7 innervation pruning in repo-Gal4 control (p = 6.535 × 10^−11^) and driving MMP-2 RNAi (p = 6.551 × 10^−11^), but no significant pruning with MMP-1 RNAi (p = 0.523). All data points with mean ± SEM. Significance: p < 0.0001 (****) and p > 0.05 (not significant, ns). The data underlying this Figure can be found in S1 Data. (TIFF) [file pbio.3003524.s007.tiff]
